# Two new genera of songbirds represent endemic radiations from the Shola Sky Islands of the Western Ghats, India

**DOI:** 10.1186/s12862-017-0882-6

**Published:** 2017-01-23

**Authors:** V.V. Robin, C. K. Vishnudas, Pooja Gupta, Frank E. Rheindt, Daniel M. Hooper, Uma Ramakrishnan, Sushma Reddy

**Affiliations:** 1 National Centre for Biological Sciences, TIFR, Bellary Road, Bangalore, 560065 India; 2 Present address – Indian Institute of Science Education and Research Tirupati, Mangalam Tirupati, 517507 India; 3 Avian Evolution Lab, Department of Biological Sciences, Faculty of Science, National University of Singapore, Singapore, 117543 Singapore; 4 Committee on Evolutionary Biology, University of Chicago, Chicago, IL 60637 USA; 5 Biology Department, Loyola University Chicago, Chicago, IL 60660 USA

**Keywords:** Phylogenetics, Birds, Shola, Passerine, Montane, Sky-islands, Taxonomy, Tropics

## Abstract

**Background:**

A long-standing view of Indian biodiversity is that while rich in species, there are few endemics or in-situ radiations within the subcontinent. One exception is the Western Ghats biodiversity hotspot, an isolated mountain range with many endemic species. Understanding the origins of the montane-restricted species is crucial to illuminate both taxonomic and environmental history.

**Results:**

With evidence from genetic, morphometric, song, and plumage data, we show that two songbird lineages endemic to the Western Ghats montane forest each have diversified into multiple distinct species. Historically labeled as single species of widespread Asian genera, these two lineages are highly divergent and do not group with the taxa in which they were previously classified but rather are distinct early divergences in larger Asian clades of flycatchers and babblers. Here we designated two new genera, the Western Ghats shortwings as *Sholicola* and the laughingthrushes as *Montecincla*, and evaluated species-limits to reflect distinct units by revising six previously named taxa and describing one novel species. Divergence dating showed that both these montane groups split from their Himalayan relatives during the Miocene, which is coincident with a shift towards arid conditions that fragmented the previously contiguous humid forest across peninsular India and isolated these lineages in the Western Ghats. Furthermore, these two genera showed congruent patterns of diversification across the Western Ghats Sky Islands, coincident with other climatic changes.

**Conclusion:**

Our study reveals the existence of two independent endemic radiations in the high montane Western Ghats or Shola Sky Islands with coincident divergence times, highlighting the role of climate in the diversification of these ancient lineages. The endemic and highly divergent nature of these previously unrecognized species underscores the dearth of knowledge about the biogeography of the Asian tropics, even for comparatively well-known groups such as birds. The substantial increase in the diversity of this region underscores the need for more rigorous systematic analysis to inform biodiversity studies and conservation efforts.

**Electronic supplementary material:**

The online version of this article (doi:10.1186/s12862-017-0882-6) contains supplementary material, which is available to authorized users.

## Background

The Western Ghats (WG), an isolated coastal mountain chain in the southwest of India, is a global biodiversity hotspot [1]. Despite a long history of human occupation, knowledge of this biodiversity remains poor [2, 3]. Modern systematic analyses to assess species distinctiveness and their responses to past climatic events are urgently needed to inform conservation efforts in such montane tropical systems as the Western Ghats, where diversity and threat levels are high [1, 4, 5].

In most current avian taxonomic treatments, WG lineages are a subset of the diverse avifaunal groups in the Himalayas and Southeast Asia. WG endemics are usually circumscribed as single species of larger Asian groups with limited differentiation across the WG mountain range [6–8]. Contrary to this traditional view, the hitherto first and only phylogenetic investigation of a WG endemic songbird challenged both these ideas – it revealed considerable genetic divergence between populations across the mountain range as well as from its congeners, rendering the traditional genus non-monophyletic [9]. The Indian subcontinent underwent dramatic climatic changes during the Cenozoic, which may have influenced species dispersal to and diversification within the WG [10, 11]. The long-favored ‘Satpura Hypothesis’ [12–16] suggested a specific colonization route for species to disperse from the Himalayas to the WG through the Satpura Hills, a narrow band of wet forest across central India. In the Miocene, peninsular India was much more humid with near continuous forest cover and since this period, changing climate conditions and local tectonic events led to gradual drying of northwestern and central India and the establishment of the distinct patches of wet-zone forested regions on the highlands [15, 17]. Until now, support of this hypothesis was mainly from observations of avian species distributions [6] due to the dearth of phylogenetic analyses of Indian birds [3].

The peaks of the WG range host a unique form of tropical montane cloud forest known as Shola, a natural matrix of forests and grasslands [18]. The Shola habitat is restricted to the highest elevation zone and is characterized by high rainfall, humidity and low temperatures relative to lower elevations [18, 19]. A variety of endemic taxa are exclusively found in this habitat [18, 19]. These species often have disjunct distributions across the WG mountain-tops, which have been likened to ‘islands’ of specific habitat and microclimatic conditions in an ‘ocean’ of unsuitable habitat [20]. While there are several classic examples of speciation and adaptive radiations across oceanic islands, only a few studies have examined similar patterns across montane or sky islands [2].

Two endemic avian species groups in the Western Ghats sky islands, the Kerala or Black-chinned Laughingthrushes (formerly placed in *Garrulax*, currently *Trochalopteron* [8, 21] or *Strophocincla* [7, 22]) and the Western Ghats Shortwing (alternatively placed in *Brachypteryx* [8, 21], *Myiomela* [7, 23] or *Callene* [24]) have been surrounded by taxonomic confusion, complicating studies of their diversity. The laughingthrushes exhibit striking plumage variation across the different isolated sky island peaks and have been alternatively considered as one [6, 25], two [7, 8, 21, 22], or four [26] species. The shortwings have been described as one [6, 21, 25] or two [7, 8] species. Previous phylogenetic analysis for some populations of WG Shortwings showed deep divergences across the sky island complex [9].

Elucidating the origins of montane species is crucial to illuminate the evolutionary and environmental history of this landscape [2]. We reconstructed the phylogenies of the WG laughingthrush and shortwing complexes to determine their evolutionary history and test hypotheses of diversification in relation to past climatic events. Furthermore, we examined discrete variation across populations of these two lineages using multiple types of data – genetic, song, plumage and morphometric – to determine species-limits and assess differentiation within the WG range.

## Methods

### Sample collection

From January 2012 to May 2013 we conducted expeditions to survey and collect samples across the entire distributional range of both species complexes (Additional file 1: Figure S1). We followed Robin et al. [9] for field sampling techniques to capture birds with multiple 12m * 2 m mist-nets and collect blood samples from the brachial vein in Queen’s lysis buffer. For one location where field sampling proved difficult (Bababudan Hills) for the shortwings, we used two museum samples from the Natural History Museum (Tring, UK; NHMUK) for DNA analysis.

### DNA sequencing

We extracted DNA (using the Qiagen Blood and Tissue Extraction Kit) and used standard procedures [9, 27] to sequence 26 and 31 individuals of the laughingthrush and the shortwing complexes, respectively, across their entire distributions (Additional file 1: Tables S1 and S2). We generated sequence data from five and eight loci, respectively, for both groups to match published phylogenies of their relatives [27–29]. For the laughingthrushes, we sequenced 5 loci: cytochrome b (CYTB), NADH dehydrogenase subunit-3 (ND3), the fifth intron of nuclear b-fibrinogen (FIB5), the third intron of the muscle-specific kinase Gene (MUSK), and the fifth intron of transforming growth factor β2 (TGF) using standard primers [30] and standard PCR procedures (see [9, 30, 31]). For shortwings, we sequenced 8 loci: NADH dehydrogenase subunit-2 (ND2), ND3, CYTB, cytochrome c oxidase 1 (CO1), intron 2 of myoglobin (MYO), introns 6 and 7 of ornithine decarboxylase (ODC), intron 11 of the glyceraldehyde-3-phosphodehydrogenase (GAPDH), and intron 3 of lactate dehydrogenase (LDH). DNA sequences were assembled, annotated, and aligned using Geneious 6.1.4.

### Phylogenetic analyses

#### WG laughingthrushes

To reconstruct the evolutionary relationships of the WG Laughingthrushes, we re-analyzed a larger clade encompassing laughingthrushes in general (Leiothrichidae) as per recent studies on sylvioid songbirds [27, 32, 33]. We assembled a matrix by incorporating representatives of key members of other laughingthrush species with our data from all lineages found in the Western Ghats. As outgroups, we included one representative of each of the other major clades of babblers [27]. We assembled two matrices of five loci each: to examine divergences within the WG Laughingthrush complex, we included all 26 WG individuals and several other babblers as outgroups; and to examine the relationships of the WG Laughingthrushes with other babblers, we assembled a matrix comprising 73 taxa (Additional file 1: Table S1). PartitionFinder v1.01 [34] determined that the best partitioning scheme of gene regions divided by rates of evolution was four partitions: 1st position CYTB + 1st position ND3, 2nd position CYTB + 2nd position ND3, 3rd position CYTB + 3rd position ND3, FIB5 + MUSK + TGF.

#### WG shortwings

Preliminary analyses found that shortwings belong within the flycatcher/chat complex. To examine variation within the WG Shortwings, we assembled a phylogenetic matrix of 34 taxa for four loci: ND2, ND3, CYTB, CO1 (Additional file 1: Table S2). To examine their placement in a broader phylogenetic context, we compiled data for species across a larger clade based on other published studies [28, 29, 35] and included all distinct WG Shortwing lineages for a total of 96 species for six loci -- ND2, CYTB, MYO, ODC, GAPDH, and LDH (Additional file 1: Table S2). The best partitioning scheme for this dataset according to PartitionFinder was 8 partitions: 1st position CYTB, 2nd position CYTB, 3rd position CYTB, 1st position ND2, 2nd position ND2, 3rd position ND2, MYO, and GAPDH + LDH + ODC.

For both groups, we conducted maximum likelihood (ML) phylogenetic analyses using RAxML 8 [36] by partitioning genes according to similar evolutionary rates determined with PartitionFinder. We conducted 1000 random bootstrap replicates that were subsequently used to search for the best ML tree. We compared relationships from this analysis to those using different optimality criteria such as maximum parsimony, using PAUP* [37], and Bayesian inference, using MrBayes 3.2 [38]. In PAUP*, we treated all characters with equal weights and ran heuristic searches of 1000 random addition replicates. In MrBayes, we used the same partition scheme as ML analyses and ran two Monte Carlo Markov Chain runs of four chains each for 20 million generations, sampling every 500th generation. We used default priors and unlinked parameters across partitions except for branch length calculations. We assessed convergence and stationarity of runs using Tracer v1.6 [39] and AWTY [40], discarding the first 5000 generations of each run as burn-in.

### Divergence dating

We used BEAST v1.75 [41] to estimate the timing of lineage divergence. For both groups, we used one representative of each distinct lineage for divergence dating analysis. In each analysis, we unlinked substitution and clock rates, and linked tree models for each locus. To calibrate the laughingthrush/babbler phylogeny, we used a secondary calibration for the divergence time between the families Timaliidae, Pellorneidae + Leiotrichidae as 20.92 Ma (standard deviation of 2.11 Ma), taken from [29], a recent time-calibrated phylogeny of Asian passerines using 13 corroborated clade ages based on fossil and biogeographic calibrations (see Additional file 1 in [29]). To calibrate the shortwing/flycatcher tree, we used the same source to date the split between the families Muscicapidae and Turdidae as 21 Ma (standard deviation of 2 Ma) [29]. We understand that dates for the bird tree of life can be controversial but we chose this source [29] because it was the most densely-sampled, recent phylogeny for taxa relevant to this study. For each analysis, we used an uncorrelated lognormal relaxed clock model with a birth-death speciation tree prior. We conducted two runs of 20 million generations, sampling every 1000th and discarding the first 5000 as burn-in. We used Tracer v1.6 [39] to ensure stationarity of chains for all parameters (ESS values >200).

### Ancestral area reconstruction

We used Lagrange v. 20130526 [42] to reconstruct ancestral areas for both groups. For laughingthrushes and relatives, we defined eight areas – Africa, Peninsular India, Himalayas, Southeast Asia, China, Sundaland, Philippines, and Assam Hills. For shortwings and relatives, we defined nine areas based on the geographic extent of these species: Africa + Southwest Asia, Peninsular India, Himalayas, Southeast Asia, China, Sundaland, Philippines, Eurasia, and Australasia/New World. In Lagrange, we assigned the ranges of each species based on their current geographical distributions. We allowed the ancestral area reconstructions to include any combination of areas. We used the time-calibrated tree from our BEAST analysis to reconstruct the likelihood of ancestral changes in distribution.

### Species limits

We conducted focused mist-net based sampling over 3 years (2011 – 2014) and several years (2000 – 2010) of mixed sampling effort (point counts, transects, *ad libitum* observations, and mist-net sampling). During this period, we captured and examined 359 WG laughingthrushes and 430 WG shortwings. We collected a variety of vocal, plumage, morphometric and genetic data, along with extensive natural history observations of these species.

To assess species limits, we compared all population clades to determine statistically discrete units of nucleotide substitutions as well as distinct differences in plumage characteristics, morphometric variation, and song features. We chose this method because it allows using all available evidence to infer absence of gene flow and to delineate evolutionarily distinct units as species. Our species delineations are consistent with multiple species concepts - phylogenetic species concept, biological species concept, general lineage concept, and integrative taxonomy.

#### Coalescent-based test of species delimitation

We utilized a coalescent-based approach to statistically test species delimitation using the program BPP v3.1 [43]. This method employs the multispecies coalescent to compare different models of species delimitation and phylogeny in a Bayesian framework while accounting for incomplete lineage sorting due to ancestral polymorphism and gene tree-species tree conflicts [43]. We used the joint species delimitation and species tree analysis in BPP to test if the number of distinct units/species as delineated by our character-based analysis (see below) was statistically significant. For each lineage, we used the genetic dataset assembled to examine within-group variation and designated individuals by population/clade. For population size parameters, we assigned the gamma prior G (2, 1000), with mean 2/2000 = 0.001. We ran each analysis at least twice to confirm consistency between runs. Each run was for 100,000 samples, sampling frequency was 5, and the burnin was set to 20,000.

#### Morphometrics

All captured birds were banded and measurements of the length of right tarsus, flattened chord right wing, bill (from base of skull) and tail were taken following SAFRING manual [44] and USDA [45] guidelines. Each measurement of a feature was repeated thrice to examine measurement error [46]. Tarsus and bill measurements were taken with Mitutoyo ABS Digimatic Caliper (Mitutoyo Corp Japan) with accuracy of 0.02mm. Wing and tail measurements were taken with a wing rule (WING15ECON Avinet Inc.) that had a flush stop and calibration from both directions.

We analyzed the four morphometric variables (bill length, tarsus length, wing length and tail length; Additional file 1: Tables S3, S4) using multivariate statistics. For each species complex, we considered each distinct population clade separately and built a linear discriminant function with a common covariance matrix for all groups (JMP version 8). This was visualized with a canonical plot including a 95% confidence ellipse of the mean of each group and biplot rays indicating the direction of variables in canonical space. The groups with non-overlapping ellipses are considered different in morphology with the direction of biplot rays indicating the variables contributing to the observed differences. Wing and tail lengths for WG Shortwings were found to be collinear, but no significant collinearity among morphometric variables was detected in WG Laughingthrushes. However, a repetition of the discriminant analyses with reduced data set of three principal components (comprising 89.76% of the variation for WG Shortwings, 85% for WG Laughingthrushes) did not alter the results, hence we retained the analyses with the original variables for easier interpretation.

#### Plumage

For plumage comparisons, we examined and noted features of each population by studying live birds (sampled in the field), photographs of sampled birds and museum specimens (Bombay Natural History Society; BNHS, India) to make side-by-side comparisons, and descriptions in the literature. We noted differences in coloration across discrete feather patches to assess whether populations had unique plumage patterns.

#### Song

Field recording of 38 individual singing males was carried out between 0700 to 1100 h following Robin et al. [47]. Each recording consisted of only one continuous song bout from a single individual recorded using a Sennheisser shotgun microphone (ME66-K6) and Marantz Digital Audio Recorder (PMD660). Recordings were converted into spectrograms in Raven Pro 1.3 [48] at a sampling rate of 48 kHz. For each song, we collected data on frequency and note length parameters for each note, with multiple songs per individual and multiple individuals per population. A detailed statistical analysis of songs and syllables across multiple populations is presented in Purushotham and Robin [49].

## Results

### Phylogenetic analyses

**Fig. 1:**
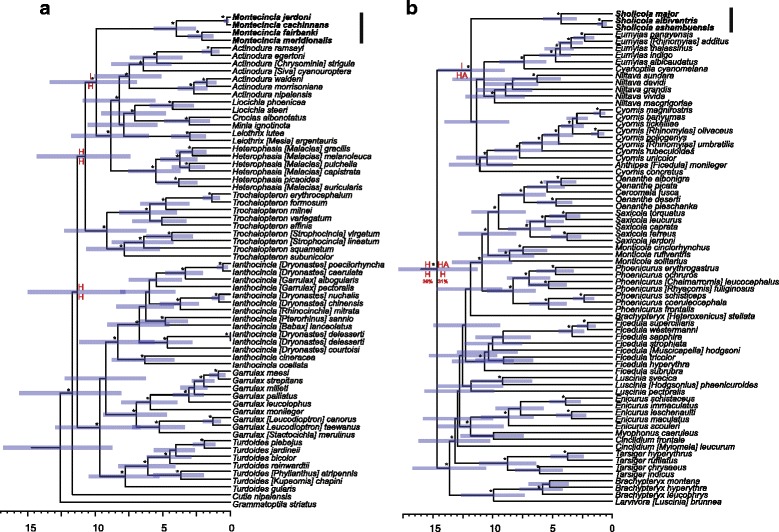
Time-calibrated phylogenies of the (**a**) laughingthrush/babbler and (**b**) shortwing/flycatcher clades. The WG Laughingthrushes, *Montecincla*, did not group with any of the other laughingthrush clades and are sister to a clade of birds traditionally not included in laughingthrushes. The WG Shortwings, *Sholicola*, are not part of shortwings (*Brachypteryx* or *Myiomela*) but are sister to a clade of Asian blue flycatchers, the Niltavinae. Generic designations follow *Clements Checklist* [8]; *Handbook of the Birds of the World* [22] names are shown in brackets if different. Node bars show 95% HPD estimates of divergence dates and stars indicate ML bootstrap of 70% or higher and Bayesian posterior probability of 95% or higher. Letters (in red) show ancestral area reconstruction at relevant nodes (I = Peninsular India; H = Himalayas; A = Southeast Asia)

The larger, family-level phylogenetic analyses indicated that both WG lineages diverged early within their respective clades (Fig. 1). Each of these two lineages is a distinct clade not closely related to the genus historically or currently grouped within. Additionally, both were found to be discrete clades deeply divergent from their closest relatives. The WG Laughingthrushes did not group with any of the other traditional laughingthrush clades (including ones designated as *Trochalopteron* or *Strophocincla*), but instead were sister to a clade composed of *Heterophasia, Minla, Actinodura, Leiothrix, Liocichla* and *Crocias* (Fig. 1a). Similarly, we reconstructed the WG Shortwings as sister to a newly uncovered clade comprised of several genera of mainly Asian blue flycatchers, the Niltavinae [28, 35], and not closely related to traditional shortwings in the genera *Myiomela* or *Brachypteryx* (Fig. 1b).

Both WG lineages are not only divergent from their closest relatives, they are also not sister to any single genus as defined under current taxonomic treatments. Thus, they cannot easily be lumped or subsumed under an existing clade without an extensive reorganization of multiple genera. As a better alternative, we designated new genus names: Western Ghats Laughingthrushes as *Montecincla* and Western Ghats Shortwings as *Sholicola* (see below for descriptions), to recognize their distinctiveness and to avoid confusion with older names associated with other taxa that are not part of these new groups.

The ancestral area reconstruction showed dispersal into the WG for *Montecincla* as most likely from the Himalayas and for *Sholicola* from the Himalayas + Southeast Asia (Additional file 1: Figures. 1, S2, S3). The two WG lineages have a similar estimated timing of separation from their closest relatives (Table 1). These dates also coincide with climatic events that led to the drying of peninsular India (Table 1), perhaps leading to discontinuing gene flow and a differentiation between peninsular forms from Himalayan / Southeast Asian forms. Furthermore, the divergence of populations within both lineages across the sky islands exhibits similar timing of diversification in response to the same biogeographic barriers (see below).

**Table 1:** Estimates of divergences dates and geological events

Event	Timing estimates (Ma)
*Montecincla* vs. *sister group* - split WG/Himalayas	11.57 (14.65 - 8.70)
*Sholicola* vs. *sister group* - split WG/Himalayas	11.83 (14.59 - 9.00)
within *Montecincla* - split across Palghat Gap [AB,CD]	4.7 (6.34 - 3.19)
within *Sholicola* - split across Palghat Gap [AB,CD]	4.33 (5.79 - 2.98)
within *Montecincla* - split across Shencottah Gap [C,D]	2.51 (3.60 - 1.60)
within *Sholicola* - split across Shencottah Gap [C,D]	0.86 (1.24 - 0.49)
within *Montecincla* - split across Chaliyar Valley [A,B]	0.33 (0.55 - 0.15)
Himalayas - peak constructional phase [55]	15 - 10.5
Tibetan Plateau - significantly uplift [55, 66]	10 - 8
Climate - enhanced aridity of Asia; onset of Indian monsoons [56–58, 66]	9 - 8
Vegetation - C4 plants start to replace C3 [56, 57]	6
Climate - major global shift; monsoons weakened [58]	2.6

### Species limits

**Fig. 2:**
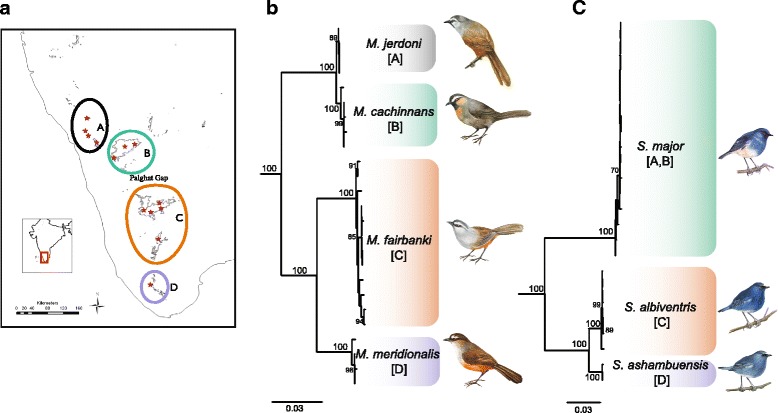
Ranges (**a**) and phylogenetic relationships of *Montecincla* (**b**) and *Sholicola* (**c**) species. **a** Inset map of the Western Ghats shows sampling localities (stars), and divisions of the sky islands based on differentiated taxa. **b & c** ML bootstrap values are shown at nodes. Bird illustrations by Maya Ramaswamy

Phylogenetic analysis of both groups showed clades corresponding to four major biogeographic regions in the WG, north to south: A- Bababudan & Banasura hills; B- Nilgiri hills; C- Anamalai, Palani, and Meghamalai hills; D- Ashambu hills (Fig. 2). *Montecincla* was comprised of four reciprocally monophyletic clades (Fig. 2b). Bayesian species delimitation using BPP also showed that these four clades were distinct species. Elevating former subspecies names, these are: *Montecincla jerdoni* (range A)*, M. cachinnans* (B)*, M. fairbanki* (C)*, M. meridionalis* (D). Similarly, analysis of *Sholicola* revealed three reciprocally monophyletic clades (Fig. 2c) that were distinct species according to BPP. While two existing subspecies names that match these units can be elevated, namely *Sholicola major* (range A, B) and *S. albiventris* (C), the third (D) is a new species we designate below. Analyses of fixed characters of plumage, morphometrics and song data (below) match the distinct units uncovered in the phylogenetic and species delimitation analyses. Therefore multiple lines of data support four species in the *Montecincla* complex and three species in the *Sholicola* complex.

#### Plumage

Our analysis of plumage variation for *Montecincla* supports four distinct groups (Table 2). All four species have distinguishing features in multiple color patches that separate these evolutionary units. Within-species variation is low and populations with similar features occur in geographically clustered sky islands (shown in Fig. 2). In *Sholicola*, *S. major* (A, B) showed striking differences from the other two species south of the Palghat Gap. The differences in plumage of between *S. albiventris* (C) and *Sholicola* sp. nov. (D; see below) are mainly in terms of the extent of white coloration on the belly. The newly described species has a considerably larger white belly patch (~24mm in length) than *S. albiventris* (~19mm) (Table 3).

**Table 2:** Plumage differences between species of *Montecincla*

Plumage feature	*M. jerdoni*	*M. cachinnans*	*M. fairbanki*	*M. meridionale*
Crown	Slaty brown	Slaty brown	Dark brown almost black	Grey brown
Chin	Black	Black	Grey	Pale grey
Ear coverts	Greyish white	Pale rufous	Pale grey	Brownish grey
Supercilium	White, long, reaching behind eye; black eye strip from lore	White, long reaching behind eye; black eye strip from lore	White, Long, extending behind eye; black stripe below lores through eyes	White, short, not extending behind eye
Nape	Slaty brown	Ashy brown	Brownish grey	Pale brown
Upper parts	Olive brown up to tail	Olive brown	Olive brown	Dull grey at nape brownish towards rump
Breast	Grey, with faint streaks	Bright rufous	Pale grey with faint streaks	Whitish grey with prominent dark streaks
Belly	Olive brown but centre of belly pale rufous	Ochraceous	Rufous to chestnut	White at centre with mild dark streaks, sides deep chestnut
Centre of belly	Pale rufous	Rufous	Rufous to chestnut	White centre
Flanks	Olive brown	Olive brown	Rufous to chestnut	Reddish chestnut

**Table 3:** Plumage differences between species of *Sholicola*

Plumage feature	*S. major*	*S. albiventris*	*S. ashambuensis*
Crown	Slaty blue	Slaty blue	Slaty blue
Supraloral stripe	Faint blue	Whitish blue	Faint blue
Throat	Slaty blue	Slaty blue	Greyish blue
Breast	Slaty blue	Slaty blue	Greyish blue
Belly	Broad white patch	Narrow white patch from centre to vent	Narrow white patch extending anteriorly from vent to breast
Flanks	Pale rufous	Greyish blue	Greyish blue
Undertails coverts	Pale rufous	White	White
Upper parts	Slaty blue	Deep blue	Pale, slaty blue

#### Morphometrics

**Fig. 3:**
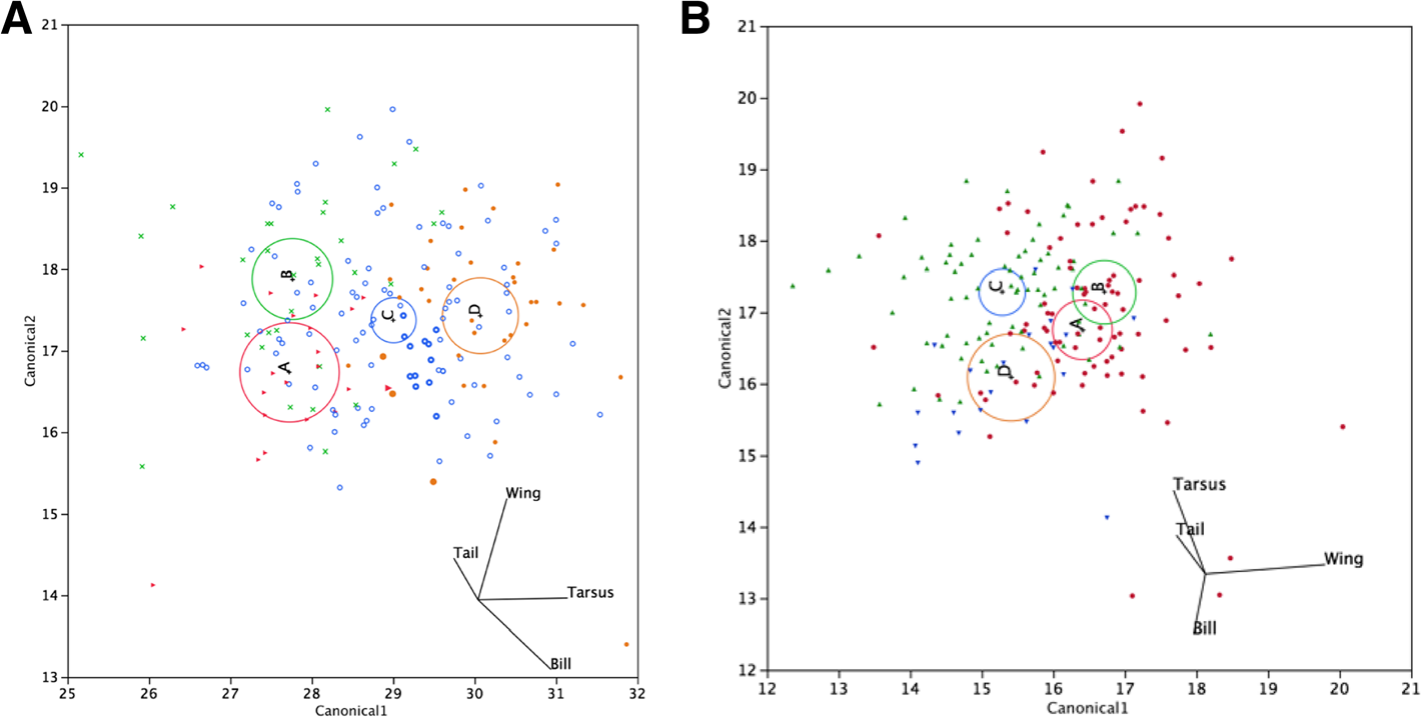
Discriminant Function Analysis showing morphometric differentiation between **a**) *Montecincla* and **b**) *Sholicola* populations/species. A-D labels refer to geographic regions (as in Fig. 2). Circles represent 95% confidence ellipses of the mean of each group, which are significantly different when not overlapping. The direction of the biplot rays show how variables contributed to the observed differences

The four species of *Montecincla* and the three species of *Sholicola* differed significantly from each other in morphospace occupied (Fig. 3). This differentiation within both *Montecincla* and *Sholicola* was largely driven by tarsus length, although the direction of increase was reversed – *Montecincla* in the southernmost region of Ashambu Hills (D) is larger than the other species further north, while in *Sholicola* the species in the southern hills were the smallest (Additional file 1: Tables S3, S4).

#### Song

**Fig. 4:**
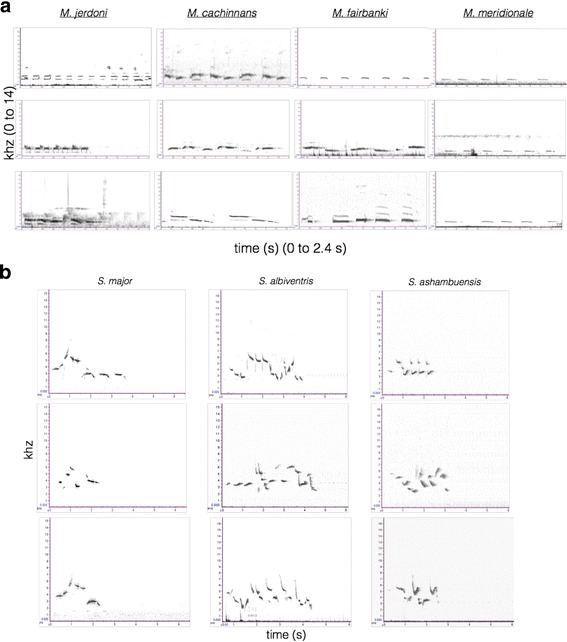
Spectrograms showing song variation across **a**) *Montecincla* and **b**) *Sholicola* species. Songs of three different individuals from each species are shown

Spectrograms reveal the presence of unique song types in all four species of *Montecincla* and all three *Sholicola* species (Fig. 4). All four species of *Montecincla* show distinct features in song. *Montecincla fairbanki* sings at a higher bandwidth and song rate than the other populations, whereas *M. meridionalis* sings at a lower bandwidth than other species, while *M. jerdoni* has a higher song complexity with longer phrases than the other populations (Additional file 1: Table S5). Songs of all *Sholicola* species are also quantitatively distinct, with differences in song length and frequency across different species (more details in Purushotham and Robin [49]). The song of the newly described species of *Sholicola* is shorter in length and higher in frequency from *S. albiventris* (Additional file 1: Table S6).

### Naming new taxa

As outlined above, the results of our analysis warrant new names for two genera and one species, in addition to elevating six subspecies to species level.

#### Western Ghats laughingthrushes

The laughingthrushes of the Western Ghats have been treated as part of the genus *Garrulax* Lesson, 1831 by Ali and Ripley [6] and as *Strophocincla* Wolters, 1981 by Rasmussen and Anderton [50], which was followed by others [22, 26]. However, in most global avian checklists, they are currently placed in *Trochalopteron* [8, 21] following phylogenetic insights [27]. Genus-level treatments in laughingthrushes have clearly been very unstable given extensive morphological variation and labile trait evolution in these birds. Our phylogenetic results reveal that all previous genus designations are inappropriate for Western Ghats Laughingthrushes and here we erect a new genus to recognize their distinctiveness.

Furthermore, we propose elevation of the four subspecies to full species level. This fits assertions by others: Rasmussen and Anderton [50] tentatively delimited two species (one north and the other south of the Palghat Gap with two subspecies each) but suggested that the four allopatric populations may each warrant species-level status with more evidence. Additionally, Praveen and Nameer [26] conducted an analysis of plumage differences and suggested that this complex was best represented as four distinct species. In line with our results of multiple character systems, we hereby propose elevation of these subspecies as four full allopatric species (Additional file 1: Figure S4). This is essentially a revival of the taxonomic treatment at the time of the original descriptions of these taxa.

*Montecincla* genus novum

Order Passeriformes: Family Leiothrichidae

Suggested common name: Chilappan

Type species: *C. [=Crateropus] cachinnaus* [sic] Jerdon, 1839 = *Strophocincla cachinnans = Montecincla cachinnans* comb. nov.

Additional species included: *Garrulax* (?) *Jerdoni* [sic] Blyth, 1851 = *Strophocincla cachinnans jerdoni* = *Montecincla jerdoni* comb. nov.; *Trochalopteron Fairbanki* Blanford, 1869 = *Strophocincla fairbanki* = *Montecincla fairbanki* comb. nov.; and *Trochalopterum meridionale* Blanford, 1880 = *Strophocincla fairbanki meridionalis* = *Montecincla meridionalis* comb. nov.

ZooBank Registry: urn:lsid:zoobank.org:act:88B21D13-2639-4BFD-8729-74308F609E4E

Diagnosis: *Montecincla* gen. nov. can be differentiated from other genera of traditional laughingthrushes - *Garrulax; Ianthocincla* Gould, 1835; *Trochalopteron*; and from *Turdoides* babblers by the combination of a prominent white supercilium, olive brown upperparts, small size, brown wings, rufous flanks and dark bill. *Montecincla* lacks the complex upperwing colouration (consisting of strong barring or conspicuous wing panels) of *Actinodura* Gould, 1836, and of *Liocichla* Swinhoe, 1877. *Montecincla* also lacks the slender greyish body and long tail of sibias of the genus *Heterophasia* Blyth, 1842. *Laniellus* Swainson, 1832 (*=Crocias* Temminck, 1836) has a diagnostic whitish underside with bold, streaked flanks that are absent in *Montecincla. Minla* Hodgson, 1837 is a genus of small birds (14cm) with a colourful reddish tail or rump, while *Montecincla* is larger (20cm) with a dull olive-brown tail and rump. *Leiothrix* Swainson, 1832, is characterized by a colourful bill and a yellow throat, contrasting with the black bill and greyish-white or black throats of *Montecincla.*

Description: *Montecincla* chilappans are medium-sized (~20cm) songbirds with rounded tails. Their upperparts are olive grey and they have a prominent white supercilium. The breast is greyish-white in three species and rufous in one (Table 2).

Etymology: *Montecincla*, of feminine gender, is a combination of Latin “mons” (gen. “montis”), with the meaning “mountain”, and Greek “kinklos”, denoting an unidentified type of songbird (often assumed to be a thrush). We chose this appropriate moniker because the genus is confined to the higher mountains of the Western Ghats. Our suggested vernacular name “Chilappan” stems from the local name of this genus (in Malayalam language), denoting their joyful cackling calls.

Comments: We suggest Banasura Chilappan as a common name for *Montecincla jerdoni* in recognition of Mt. Banasura, one of its strongholds and its type locality. Similarly, we suggest the common names Nilgiri Chilappan for *Montecincla cachinnans*, Palani Chilappan for *Montecincla fairbanki*, and Ashambu Chilappan for *Montecincla meridionalis*.

#### Western Ghats blue robins or shortwings

The species were historically placed in the genus *Phoenicura* Swainson, 1831, later in *Callene* Blyth, 1847, then in *Brachypteryx* Horsfield, 1821 and more recently in *Myiomela*. None of these generic names are appropriate given our phylogenetic analysis. We thus erect a new genus to recognize the distinctness of Western Ghats Blue Robins (also known as Western Ghats Shortwings). In addition, we recognize three distinct species in this genus, including one new, previously unrecognized species.

*Sholicola* genus novum

Order Passeriformes: family Muscicapidae

Suggested common name: Sholakili

Type species: *Phoenicura major* Jerdon, 1841 = *Myiomela major = Sholicola major* comb. nov.

Additional species included: *Callene albiventris* Blanford, 1868 = *Myiomela albiventris = Sholicola albiventris* comb. nov.; *Sholicola ashambuensis* Robin, Vishnudas, Rheindt, Gupta, Hooper, Ramakrishnan & Reddy (see below).

ZooBank Registry: urn:lsid:zoobank.org:act:66837381-635E-476C-9454-092BB03B0A91

Diagnosis: *Sholicola* is sexually monomorphic, thus differing from the following closely related and sexually dimorphic genera: *Cyornis* Blyth, 1843; *Eumyias* Cabanis, 1850; *Niltava* Hodgson, 1837; and *Cyanoptila* Blyth, 1847. Being largely blue, it differs from *Anthipes* Blyth, 1847, which comprises brown birds with distinctive white throats. Members of *Sholicola* are resident birds with no known seasonal migration as in members of *Niltava* and *Cyanoptila.* The three species of *Sholicola* are phylogenetically distant from the genera they were previously placed under (*Brachypteryx*, *Callene, Phoenicura, Myiomela*). They also differ from these genera in having a sexually monomorphic plumage dominated by blue colouration.

Description: *Sholicola* is a genus of terrestrial blue flycatchers confined to high altitude (above 1200m) forests in the Shola-grassland complexes of the Western Ghats. Their general plumage is dominated by blue. They have a bluish-white band above the black lores, a slightly curved bill tip, well-developed rictal bristles, a short, nearly square tail, and long tarsi. The birds are mainly restricted to the understory, rarely venturing above three meters and feeding on insects singly or in pairs. There is no known sexual dimorphism in plumage though morphometric differences in wing length are recognized [10].

Etymology: *Sholicola*, of masculine gender, is a combination of Shola (the local name for montane forests in the Western Ghats) and the suffix –cola (from the Latin verb “colere”), meaning “dweller". We also suggest the common English name Sholakili, where -kili is the local name for “bird”.

Description of a new species in the genus Sholicola:

*Sholicola ashambuensis*, species nova

English name: Ashambu Sholakili

Holotype: Trivandrum Museum of Natural History (TMNH) No. 725, collected by H.S. Fergusson on 3 May 1903 in the Chemunji Hills, Travencure (Travancore).

Etymology: The adjective *ashambuensis* denotes the species’ geographical locality, the Ashambu Hills of southern India.

ZooBank Registry: urn:lsid:zoobank.org:act:CB9F32BC-750B-48AA-8F87-C9727144019D

Diagnosis: The following characters can be used to diagnose *Sholicola ashambuensis*.

1. Despite being smaller in general body size, *S. ashambuensis* has a considerably larger white belly patch (~24mm in length) than *S. albiventris* (~19mm). Only one *S. ashambuensis* specimen was available for measurements.
2. *S. ashambuensis* is a smaller bird (Additional file 1: Figure S5) with a shorter tarsus and longer bill (Additional file 1: Table S7; Additional file 1: Figure S6) than *S. albiventris* based on an examination of 76 S*. albiventris* and 21 *S. ashambuensis.*
3. *S. ashambuensis* has a distinct song (Additional file 1: Figure S7) with a higher mean maximum frequency, but narrower song bandwidth, shorter notes and shorter song bouts than *S. albiventris*, based on 119 *S. ashambuensis* songs and 203 *S. albiventris* songs.
4. When compared side-by-side, *S. ashambuensis* is paler blue than *S. albiventris*.

In addition to these phenotypic diagnostic characters, *S. ashambuensis* forms a reciprocally monophyletic clade based on DNA data from about 25 individuals. Further population genetic data with 15 microsatellites and 218 individuals (including 17 of *S. ashambuensis*) also support significant genetic differentiation [51].

Description of holotype: A small, overall dark-blue flycatcher with a large white belly patch reaching to the vent, black lores, and a bluish-white band above the lores. The holotype measures: bill – 16mm, wing– 81mm, tail – 61mm, tarsus – 21mm.

Distribution: Ashambu hills south of Shenkottah gap, southern India, mostly above 1200m elevation (Additional file 1: Figure S4).

Comments: The subtle plumage differentiation and limited fieldwork in the Ashambu Hills, including an absence of systematic capture-based studies, are perhaps the reason why *S. ashambuensis* has gone taxonomically unrecognized until now. The only museum specimen to our knowledge, the holotype (described above), was re-discovered (by CKV) after being locked up for about 120 years in a drawer of the Travancore Museum. Other specimens thought to be from the southern range are missing (and perhaps destroyed). There are possibly two more specimens in NHMUK (Tring) based on geography, but these were not examined by the authors.

## Discussion

Using a modern systematic approach, we uncovered two deeply divergent lineages in the Indian avifauna and provided the first evidence of in-situ avian radiations within the Indian subcontinent. These new genera are not only phylogenetically distant from the clades with which they were previously classified, but also constitute lineages that diverged early from their closest relatives in larger Asian clades, many of which were only recently identified themselves. Our study underscores the importance of continued systematic studies in untangling taxonomic confusions to better understand local, regional, and global patterns of diversification.

The two WG lineages we examined, previously known as laughingthrushes and shortwings, do not belong to either of these two groups. Our results corroborate previous studies [27, 32, 33] that showed laughingthrushes to be polyphyletic. The WG Laughingthrushes do not group with any of the other traditional laughingthrush clades including species placed within *Trochalopteron* or *Strophocincla*. Rather, they are in a distinct clade of their own that is sister to several genera traditionally not considered laughingthrushes. We introduced a new genus name, *Montecincla*, to highlight the phylogenetic and biological distinction of the Western Ghats Laughingthrushes as well as to avoid confusion with alternative names. The taxonomic history of the WG Shortwings has gone through a similar level of confusion. Due to behavioral and plumage traits, they were traditionally thought to be shortwings (genus *Brachypteryx*). The first phylogenetic analysis of the WG species found that they were not closely related to *Brachypteryx* [9]. Our analysis conclusively shows that WG Shortwings are actually flycatchers and sister to a newly discovered clade of several genera of Asian blue flycatchers, the Niltavinae.

We present the first evidence of in-situ bird radiations in the Western Ghats mountains. Our examination of various character data – genetic, song, plumage and morphometric – all point to considerable differentiation among populations that we propose to comprise four species in the genus *Montecincla* and three species in the genus *Sholicola.* The complete distributions of both species complexes lie along a 400 km latitudinal gradient comprising the highest elevations, the sky islands of the Western Ghats. Further intensive examinations in this region may reveal patterns of similar endemic radiations in other taxa.

The diversification of *Montecincla* and *Sholicola* from their respective sister taxa supports the classic model of species colonizing the WG from the Himalayas. The estimated divergence dates for corresponding species on either side of common barriers are similar across these two groups, which is compelling evidence for a vicariance model. We interpret our estimated dates using the best calibration currently available and in light of the corresponding known climatic events that may have led to these biogeographic patterns. Given that there is much disagreement about the age of bird diversification (see [52–54]), we acknowledge that different divergence dating methods and calibrations may provide alternative estimates of these dates. However, the relative timings of these divergences will remain the same. Our analyses provides a compelling demonstration that these two lineages have nearly identical divergence times, regardless of the exact ages, implying a common mechanism.

The coincidence in timing of colonization of the WG by *Montecincla* (11.57 Ma) and *Sholicola* (11.83 Ma) supports a scenario of initial range expansion during a wet, cool period in the mid-to-late Miocene [15, 55, 56] that allowed these cool-adapted lineages to disperse across the moist forests that covered the Indian subcontinent followed by subsequent vicariance as the subcontinent became drier and more seasonal in the late Miocene [56–58], which likely led to local extinction in the central region and current isolation in the WG. Intriguingly, another species of laughingthrush found in the Western Ghats, *Ianthocincla* [*Dryonastes*] *delesserti*, represents a more recent colonization into this region (see Fig. 1a).

Diversification within the WG in the Pliocene and Pleistocene was likely driven by climatic events that expanded and contracted the cool montane forest in southwestern India. Within the WG, both lineages show similar divergence dates (4.7 and 4.33 Ma) across the Palghat Gap. This break in the mountain chain is a major habitat barrier for many taxa [59–61]. It is the widest and deepest valley in the range and is thought to be the result of an ancient geological fissure (~500 Myr old) [9]. The tight correspondence of divergence dates in *Montecincla* and *Sholicola* across this now dry gap indicates that suitable wet, cool habitat, which was needed for these taxa to disperse, likely only lasted for a brief period in the Pliocene. Divergences across narrower gaps are not consistent across these two lineages, or several other bird species [60]. Climatic fluctuations in the Pleistocene were more numerous and variable in strength and duration [58, 62], perhaps leading to more individual responses by species across the narrow valleys within the WG. A more thorough survey of endemic taxa and their diversification history is crucial for a better understanding of the evolutionary history and assembly of this unique ecosystem.

## Conclusions

The discovery of two independent endemic bird radiations in the Shola Sky Islands highlights the evolutionary role that such habitats can play in the diversification of lineages and the need for additional systematic studies that can potentially find other taxa with similar patterns. An important result from our findings is the dramatic increase in the biodiversity inventory of the WG, from the previously recognized three species to seven endemic species, each with much narrower ranges. Tropical sky island species are thought to be the most susceptible to anthropogenic climate change [4], and with 50% of Shola habitat in the WG already lost [5], these findings provide a much needed impetus for conservation [63]. With the Western Ghats continuing to lose between 0.57%–0.91% [64, 65] of their forest habitats each year, endemic species face extraordinary conservation challenges. Studies such as this not only clarify the taxonomic and phylogenetic information needed to quantify biodiversity but also urge the need to assess the possible responses of these species to anthropogenic climate change.

## Additional file

Additional file 1: Figure S1. Sample collection sites across the sky islands of the Western Ghats. **Figure S2.** Ancestral area analysis using Lagrange for *Montecincla.* Area codes for terminal taxa are A-Africa, B-peninsular India, C-Himalayas, D-Southeast Asia, E-China, F-Sundaland, G-Philippines, and H-Assam. The most likely ancestral range reconstruction for relevant nodes (in red) are shown. **Figure S3.** Ancestral area analysis using Lagrange for *Sholicola*. Area codes for terminal taxa are I-Peninsular India, H-Himalayas, A-Southeast Asia, D-Eurasia, E-Australasia F- Africa + Southwest Asia, C-China, S-Sundaland, P-Philippines, E-Eurasia, and N-Australasia/New World. The most likely ancestral range reconstruction for relevant nodes (in red) are shown with their relative probabilities. **Figure S4.** Map showing the species distributions of *Montecincla* chilappans and *Sholicola* sholakillis. **Figure S5.** Photographs of *Sholicola ashambuensis sp. nov.* a) holotype in the Trivandrum Museum of Natural History; b) in comparison with other members of *Sholicola*: *S. ashambuensis* (right)*,* with larger white belly contrasted with *S. albiventris* (middle), *S. major* (left). **Figure S6.** a) Discriminant Function Analysis of morphometric differences between *Sholicola ashambuensis* and *S. albiventris* based on 97 individuals shows 79.4% accurate classification and significant differences in the means (non overlapping 95% confidence interval circles); b) Box plots showing morphometric differences in bill and tarsus lengths in *Sholicola ashambuensis* and *S. albiventris*. **Figure S7.** Discriminant Function Analysis of *Sholicola ashambuensis* and *S. albiventris* songs based on 217 song recordings shows 80% accurate classification and significant differences in the means (non overlapping 95% confidence interval circles). **Table S1**: Samples used to reconstruct *Montecincla* phylogeny. **Table S2**: Samples used to reconstruct *Sholicola* phylogeny. **Table S3**: Morphometric variation in *Montecincla* species. **Table S4**: Morphometric variation in *Sholicola* species. **Table S5**: Song variation in *Montecincla* species in the Western Ghats Sky Islands. **Table S6**: Song variation in *Sholicola* species in the Western Ghats Sky Islands. **Table S7**: Comparison of morphometric measurements of *Sholicola ashambuensis* and *S. albiventris. (PDF 3728 kb)*
