# Supplementary material for: Two new genera of songbirds represent endemic radiations from the Shola Sky Islands of the Western Ghats, India
Source: BMC Evol Biol. 2017 Jan 23;17:31. doi: 10.1186/s12862-017-0882-6 (PMC5259981; doi:10.1186/s12862-017-0882-6)
Supplement: Additional file 1: Figure S1. — Sample collection sites across the sky islands of the Western Ghats. Figure S2. Ancestral area analysis using Lagrange for Montecincla. Area codes for terminal taxa are A-Africa, B-peninsular India, C-Himalayas, D-Southeast Asia, E-China, F-Sundaland, G-Philippines, and H-Assam. The most likely ancestral range reconstruction for relevant nodes (in red) are shown. Figure S3. Ancestral area analysis using Lagrange for Sholicola. Area codes for terminal taxa are I-Peninsular India, H-Himalayas, A-Southeast Asia, D-Eurasia, E-Australasia F- Africa + Southwest Asia, C-China, S-Sundaland, P-Philippines, E-Eurasia, and N-Australasia/New World. The most likely ancestral range reconstruction for relevant nodes (in red) are shown with their relative probabilities. Figure S4. Map showing the species distributions of Montecincla chilappans and Sholicola sholakillis. Figure S5. Photographs of Sholicola ashambuensis sp. nov. a) holotype in the Trivandrum Museum of Natural History; b) in comparison with other members of Sholicola: S. ashambuensis (right), with larger white belly contrasted with S. albiventris (middle), S. major (left). Figure S6. a) Discriminant Function Analysis of morphometric differences between Sholicola ashambuensis and S. albiventris based on 97 individuals shows 79.4% accurate classification and significant differences in the means (non overlapping 95% confidence interval circles); b) Box plots showing morphometric differences in bill and tarsus lengths in Sholicola ashambuensis and S. albiventris. Figure S7. Discriminant Function Analysis of Sholicola ashambuensis and S. albiventris songs based on 217 song recordings shows 80% accurate classification and significant differences in the means (non overlapping 95% confidence interval circles). Table S1: Samples used to reconstruct Montecincla phylogeny. Table S2: Samples used to reconstruct Sholicola phylogeny. Table S3: Morphometric variation in Montecincla species. Table S4: Morphometric variation in Sholic [file 12862_2017_882_MOESM1_ESM.pdf]

Electronic supplemental material for Robin et al. Two new songbird genera represent endemic radiations from the Western Ghats of India

Table S1. Samples used to reconstruct *Montecincla* phylogeny. All individuals of *Montecincla* were used for the within-species phylogeny but only the samples in bold were included in the higher-level analysis.

**Species**	**Sample Number**	**Voucher Number**	**Locality**	**Gene**				
				ND3	CYTB	TGF	FIB5	MUSK
Bankit ID					**1815018**			
M. jerdoni	**glt1**	**159101**	**Banasura**	**KR083653**	**KR083635**	**KY451860**	**KR083673**	**KR083715**
M. jerdoni	glt2	159102	Banasura	KR083661	KR083633	**KY451861**	KR083687	KR083699
M. jerdoni	glt3	159107	Banasura	KR083668	KR083636	**KY451862**	KR083688	KR083701
M. jerdoni	glt4	159189	Ambalapara	KR083665	KR083634	**KY451863**	KR083675	KR083711
M. cachinans	**glt5**	**159152**	**Sispara**	**KR083658**	**KR083631**	**KY451864**	**KR083689**	**KR083708**
M. cachinans	glt6	159153	Sispara	KR083666	KR083647	**KY451865**	KR083684	KR083697
M. cachinans	glt7	159155	Sispara	KR083655	KR083632	**KY451866**	KR083676	KR083704
M. cachinans	glt8	159156	Sispara	KR083669	KR083641	**KY451867**	KR083694	KR083703
M. cachinans	glt9	kt6	Kotagiri		KY462741			
M. cachinans	glt10	kt7	Kotagiri		KR083638			
M. fairbanki	glt11	1897	Kodi	KR083657	KR083650		KR083685	KR083698
M. fairbanki	glt12	1899	Kodi	KR083670	KR083644		KR083674	KR083705
M. fairbanki	glt13	1900	Kodi		KY462742		KR083693	
M. fairbanki	glt14	2034	Kodi		KR083651		KR083677	
M. fairbanki	**glt15**	**2095**	**Munnar**	**KR083671**	**KR083649**	**KY451868**	**KR083686**	**KR083700**
M. fairbanki	glt16	2096	Munnar	KR083662	KR083645	**KY451869**	KR083679	KR083706
M. fairbanki	glt17	2097	Munnar		KY462743		KR083690	
M. fairbanki	glt18	159201	Munnar		KR083643		KR083680	
M. fairbanki	glt19	1484	High Wavies	KR083659	KR083640	KY451870	KR083678	KR083714
M. fairbanki	glt20	2046	High Wavies	KR083652	KR083630	KY451871	KR083692	KR083702
M. fairbanki	glt21	2047	High Wavies	KR083656	KR083639	KY451872	KR083683	KR083713
M. fairbanki	glt22	2048	High Wavies	KR083667	KR083642	KY451873	KR083672	KR083710
M. meridionalis	glt23	159133	Peppara	KR083654	KR083648	KY451874	KR083695	KR083696
M. meridionalis	glt24	159134	Peppara	KR083664	KR083646		KR083681	KR083707
M. meridionalis	**glt25**	**159138**	**Peppara**	**KR083660**	**KR083637**	**KY451875**	**KR083682**	**KR083712**
M. meridionalis	glt26	159139	Peppara	KR083663	KR083629	**KY451876**	KR083691	KR083709
Ianthocincla [Dryonastes] delesserti	WLT1	74021		KY462752	KJ702948		KT002691	KM378648
Ianthocincla [Dryonastes] delesserti	WLT2	74019		KY462753	KJ702940		KT002689	KM378644
[from published studies]								
Actinodura [Chrysominla] strigula	*KUNHM 15184*		Myanmar	JN826858	JN827120	JN826343	JN826092	JN825849
Actinodura [Siva] cyanouroptera	*KUNHM 11281*		China	JN826856	JN827118	JN826341	JN826090	JN825847
Actinodura egertoni	*KUNHM 15098*		Myanmar	JN826715	JN826988	JN826213	JN825968	JN825741
Actinodura morrisoniana	*AMNH DOT5226*		Taiwan	JN826718	JN826992	JN826217	JN825971	-
Actinodura nipalensis	*AMNH DOT5594*		Nepal	JN826719	JN826993	JN826218	-	-
Actinodura ramsayi	*AMNH DOT2629*		Vietnam	JN826721	JN826995	JN826219	JN825972	JN825743
Actinodura waldeni	*CAS 95491*		China	JN826723	JN826997	JN826221	JN825974	JN825745
Alcippe brunneicauda	*KUNHM 17766*		Borneo	JN826726	JN827000	JN826224	JN825977	JN825748
Alcippe grotei	*AMNH*		Vietnam	JN826728	JN827002	JN826226	JN825978	JN825749
Alcippe morrisonia	*KUNHM 6662*		China	JN826730	JN827004	JN826228	JN825981	JN825751
Alcippe nipalensis	*KUNHM 15244*		Myanmar	JN826731	JN827005	JN826229	JN825982	JN825752
Alcippe peracensis	*AMNH*		Vietnam	JN826733	JN827007	JN826231	JN825984	JN825753
Alcippe poioicephala	*USNM 6060*		Myanmar	JN826735	JN827009	JN826233	JN825986	JN825756
Cutia nipalensis	*USNM 475768*		Vietnam	JN826748	-	JN826244	-	-
Garrulax [Leucodioptron] canorus	*KUNHM 10389*		China	JN826760	JN827023	JN826253	JN825998	JN825769
Garrulax [Leucodioptron] taewanus	*T0276*		Taiwan		FJ841474			
Garrulax [Stactocichla] merulinus	*KUNHM 15190*		Myanmar	JN826772	JN827035	JN826262	JN826008	JN825779
Garrulax leucolophus	*KUNHM 15247*		Myanmar	JN826767	JN827030	JN826258	JN826004	JN825775
Garrulax maesi	*KUNHM 10152*		China	JN826770	JN827033	JN826261	JN826007	JN825778
Garrulax milleti	*AMNH*		Vietnam	JN826774	JN827037	JN826264	JN826010	-
Garrulax monileger	*USNM 5596*		Myanmar	JN826778	JN827042	JN826268	JN826015	-
Garrulax palliatus	*KUNHM 17732*		Borneo	JN826783	JN827047	JN826273	JN826020	-
Garrulax strepitans	*3756*		captive		JF804083			
Grammatoptila striata	*USNM 15255*		Myanmar	JN826790	JN827054	JN826280	JN826027	JN825793
Heterophasia [Malacias] auricularis	*AMNH DOT5177*		Taiwan	JN826794	JN827058	JN826283	JN826029	JN825794
Heterophasia [Malacias] capistrata	*AMNH DOT5573*		Nepal	JN826796	JN827060	JN826284	JN826030	JN825795
Heterophasia [Malacias] gracilis	*USNM 5683*		Myanmar	JN826797	JN827061	JN826285	JN826031	JN825796
Heterophasia [Malacias] melanoleuca	*CAS 95600*		China	JN826800	JN827064	JN826288	JN826034	JN825799
Heterophasia [Malacias] pulchella	*KUNHM 15166*		Myanmar	JN826803	JN827067	JN826291	JN826037	JN825801
Heterophasia picaoides	*USNM 15174*		Myanmar	JN826801	JN827065	JN826289	JN826035	JN825800

**Species**	**Sample Number**	**Voucher Number**	**Locality**	**Gene**				
				ND3	CYTB	TGF	FIB5	MUSK
Ianthocincla [Babax] lanceolata	*KUNHM 11238*		China	JN826737	JN827011	JN826234	JN825987	JN825758
Ianthocincla [Dryonastes] caerulata	*4531*		captive		JF804104			
Ianthocincla [Dryonastes] chinensis	*KUNHM 10366*		China	JN826761	JN827024	JN826254	JN825999	JN825770
Ianthocincla [Dryonastes] courtoisi	*1768*		captive		JF804062			
Ianthocincla [Dryonastes] nuchalis	*USNM 15315*		Myanmar	JN826780	JN827044	JN826270	JN826017	JN825785
Ianthocincla [Dryonastes] poecilorhyncha	*KUNHM 11128*		China	JN826786	JN827050	JN826276	JN826023	JN825790
Ianthocincla [Garrulax] albogularis	*AMNH DOT5626*		Nepal	JN826759	JN827022	JN826252	JN825997	JN825768
Ianthocincla [Garrulax] pectoralis	*KUNHM 11125*		China	JN826784	JN827048	JN826274	JN826021	JN825788
Ianthocincla [Pterorhinus] sannio	*KUNHM 11172*		China	JN826787	JN827051	JN826277	JN826024	JN825791
Ianthocincla [Rhinocichla] mitrata	*LSUMNS B36460*		Borneo	FJ460844	JN827041	FJ460912	JN826014	JN825783
Ianthocincla cineracea	*KUNHM 11043*		China	JN826762	JN827025	JN826255	JN826000	JN825771
Ianthocincla ocellata	*KUNHM 11342*		China	JN826782	JN827046	JN826272	JN826019	JN825787
Leiothrix [Mesia] argentauris	*KUNHM 15203*		Myanmar	JN826820	JN827083	JN826306	JN826054	JN825814
Leiothrix lutea	*AMNH DOT6507*		Vietnam	JN826822	JN827085	JN826308	JN826056	JN825816
Liocichla phoenicea	*KUNHM 10078*		China	JN826825	JN827088	JN826311	JN826059	JN825819
Liocichla steerii	*AMNH DOT5178*		Taiwan	JN826827	JN827089	JN826313	JN826061	JN825821
Malacopteron magnirostre	*KUNHM 12358*		Borneo	JN826847	JN827110	JN826333	JN826083	JN825840
Minla [Leioptila] annectens	*USNM 336017*		Thailand	JN826745		-	-	-
Minla ignotincta	*KUNHM 11346*		China	JN826857	JN827119	JN826342	JN826091	JN825848
Pomatorhinus ferruginosus	*KUNHM 15146*		Myanmar	JN826890	JN827151	JN826372	JN826124	JN825877
Trochalopteron [Strophocincla] lineatum	*FMNH 347872*		Pakistan	JN826768	JN827031	JN826259	JN826005	JN825776
Trochalopteron [Strophocincla] virgatum	*USNM 5627*		Myanmar	JN826793	JN827057	JN826282	JN826028	-
Trochalopteron affine	*KUNHM 15099*		Myanmar	JN826758	JN827021	-	JN825996	JN825767
Trochalopteron erythrocephalum	*KUNHM 15193*		Myanmar	JN826764	JN827027	JN826256	JN826002	JN825773
Trochalopteron formosum	*AMNH DOT9336*		captive	JN826766	JN827029	JN826257	JN826003	JN825774
Trochalopteron milnei	*KUNHM 15101*		Myanmar	JN826776	JN827039	JN826266	JN826012	JN825781
Trochalopteron squamatum	*KUNHM 15191*		Myanmar	JN826788	JN827052	JN826278	JN826025	JN825792
Trochalopteron subunicolor	*USNM 15264*		Myanmar	JN826791	JN827055	JN826281	-	-
Trochalopteron variegatum	*AMNH DOT5537*		Nepal	JN826792	JN827056	-	-	-
Turdoides [Kupeornis] chapini	*AMNH 348502*		Congo	JN826819	-	-	-	-
Turdoides [Phyllanthus] atripennis	*AMNH DOT2033*		Liberia	JN826883	JN827146	JN826365	JN826118	-
Turdoides bicolor	*UWBM 70438*		South Africa	JN826969	JN827219	JN826438	JN826190	JN825943
Turdoides gularis	*USNM 5712*		Myanmar	JN826971	JN827221	JN826440	JN826192	JN825945
Turdoides jardineii	*UWBM 52803*		South Africa	JN826973	JN827223	JN826442	JN826194	JN825947
Turdoides plebejus	*KUNHM 20025*		Sierra Leone	JN826974	JN827224	JN826443	JN826195	JN825948
Turdoides reinwardtii	*FMNH 396646*		Ghana	JN826976	JN827227	JN826446	-	JN825951
Yuhina brunneiceps	*AMNH DOT5153*		Taiwan	JN826980	JN827230	JN826450	JN826200	JN825954

Table S2. Samples used to reconstruct *Sholicola* phylogeny. All individuals of *Sholicola* were used for the within-species phylogeny but only the samples in bold were included in the higher-level analysis.

**Species**	**Sample Number**	**Voucher no**	**Locality**			**Gene**					
				ND3	CYTB	ND2	CO1	MYO	ODC	GAPDH	LDH
S. major	SW.A.1	BR8(BR569)	Ambalapara	KR083727	KR083719	KY451838	KY451880				
S. major	SW.A.2	BR9(BR571)	Ambalapara	KR083737	KR083721	KY451839	KY451881				
S. major	SW.A.3	BR10(BR587)	Ambalapara	KR083736	KR083722	KY451840	KY451882				
S. major	SW.A.4	BN2/264507	Banasura	KR083726	KR083725	KY451841	KY451883				
S. major	SW.A.5	BN3/264508	Banasura	KR083731	KR083718	KY451842	KY451884				
S. major	SW.A.6	BN4/264509	Banasura	KR083729	KR083716	KY451843	KY451885				
S. major	SW.A.7	VM1/264229	Vellarimala	KR083740	KR083724	KY451844	KY451886				
S. major	SW.A.8	VM2/264230	Vellarimala	KR083743	KR083720	KY451845	KY451887				
S. major	SW.A.9	VM4/264232	Vellarimala	KR083728	KR083723	KY451846	KY451888				
S. major	**SW.B.11**	**OT1**	**Ooty**	KR083733	GU644540	KY451847	GU644540	KY462737	KY462754	GU644507	KY462739
S. major	SW.B.12	OT2	Ooty	KR083746	GU644574	KY451848	GU644541				
S. major	SW.B.13	KT4	Kotagiri	KR083732	KR083717	KY451849	KY451889				
S. major	Brmh01		Brahmagiris	KY462745			KY451877				
S. major	Brmh02		Brahmagiris	KY462746			KY451878				
S. major	Brmh03		Brahmagiris		KY462749	KY462730	KY451879				
S. major	Brmh04		Brahmagiris		KY462750						
S. major	Brmh06		Brahmagiris	KY462747	KY462751	KY462731					
S. major	Brmh07		Brahmagiris	KY462748		KY462732					
S. major	Baba01		Bababudan Hills	KY462744	GU644572	KY462729	GU644539				
S. major	1949Whil3659		Bababudan Hills	KY462735	KY462736		KY451891				
S. major	1949Whil3658		Bababudan Hills	KY462733	KY462734		KY451890				
S. albiventris	**SW.C.15**	**GR4**	**Grasshills**	KR083739	GU644578	KY451850	GU644545	KY462738	KY462755	GU644512	KY462740
S. albiventris	SW.C.16	GR5	Grasshills	KR083738	GU644579	KY451851	GU644546				
S. albiventris	SW.C.18	KD1	Kodikanal	KR083745	GU644582	KY451852	GU644549				
S. albiventris	SW.C.19	KD2	Kodikanal	KR083730	GU644583	KY451853	GU644550				
S. albiventris	SW.C.20	HW2	Highwavies	KR083742	GU644585	KY451854	GU644552				
S. albiventris	SW.C.21	HW5	Highwavies	KR083744	GU644588	KY451855	GU644555				
S. albiventris	SW.C.22	HW6	Highwavies	KR083747	GU644589	KY451856	GU644556				
S. ashambuens	**SW.D.23**	**PR2**	**Peppara**	KR083734	GU644596	KY451857	GU644563		KY462756	GU644530	
S. ashambuens	SW.D.24	PR5	Peppara	KR083741	GU644599	KY451858	GU644566				
S. ashambuens	SW.D.25	PR6	Peppara	KR083735	GU644600	KY451859	GU644567				
[from published studies]											
Anthipes [Ficedula] monileger			Nepal - Bhojpur Distr.		KJ456192	KJ455321		GU358730	GU358860	GU358995	HM633446
Brachypteryx [Heteroxenicus] stellata			China - Yunnan		KJ456303	KJ455459			KJ455810	KJ455053	KJ454702
Brachypteryx [Myiomela] albiventris					GU644586	VV01		VV01		GU644536	VV01
Brachypteryx [Myiomela] major					GU644574	VV03		VV03		GU644508	VV03
Brachypteryx hyperythra			Bengal, Neora valley		KJ456204	KJ455334					
Brachypteryx leucophrys			Indonesia - Java		HM633263	KJ455335		HM633546	HM633685		HM633405
Brachypteryx montana			Myanmar - Nat Ma Taung		KJ456205	KJ455336		GU358707	GU358835	GU358970	HM633406
Calliope [Luscinia] calliope				NC_015074	NC_015074	NC_015074	NC_015074				
Calliope [Luscinia] pectoralis			India; China		KJ456329	KJ455486		HM633603	HM633739		HM633463
Cercomela fusca					GU055451	GU055403		HM633641	HM633771	GU358974	HM633500
Cinclidium [Myiomela] leucurum			China - Yunnan		KJ456358	KJ455520		GU358718	GU358846	GU358981	HM633418
Cinclidium frontale			Bengal, Neora valley		KJ456230	KJ455378		GU358717	GU358845	GU358980	
Cochoa purpurea			India - Darjeeling		KJ456236	KJ455385		KJ454779	KJ455756	KJ455002	
Cochoa viridis					EU154587	AY752336		EU154698	EU154812		
Copsychus [Saxicoloides] fulicatus					HM633377	GU358823		GU358764	GU358894	GU359029	HM633518
Copsychus malabaricus					HM633277	DQ466859		HM120160	GU358847	GU358982	HM633420
Copsychus saularis			Bali; Nepal		HM633278	KJ455390		GU358719	GU358848	GU358983	HM633421
Cyanoptila cyanomelana				NC_015074	NC_015074	NC_015074	NC_015074				
Cyanoptila cyanomelana					DQ365019	GU358791		GU358723	HM633709	GU358987	HM633430
Cyornis [Rhinomyias] olivaceus					HM633369			HM633651	HM633781		HM633510
Cyornis [Rhinomyias] umbratilis					HM633370			HM633652	HM633782		HM633511
Cyornis banyumas					HM633287			HM633570	HM633710		HM633431
Cyornis concretus					HM633288			HM633571	HM633711		HM633432
Cyornis magnirostris			Thailand - Chiang Mai		KJ456246	KJ455398		KJ454785	KJ455766		KJ454690
Cyornis poliogenys			India - Arunachal Pradesh		KJ456247	KJ455399		KJ454786	KJ455767	KJ455013	KJ454691
Cyornis rubeculoides			Myanmar - Chin Hills		HM633290	GU358792		GU358724	GU358853	GU358988	HM633433
Cyornis tickelliae					KJ456248	KJ455400		KJ454787	KJ455768	KJ455014	KJ454692
Cyornis unicolor					HM633291	KJ455401		HM633574	HM633713		HM633434
Enicurus immaculatus			Nepal - Bhojpur Distr.		KJ456266	KJ455421					
Enicurus leschenaulti			China - Yunnan		KJ456267	KJ455422		GU358726	GU358855	GU358990	HM633435
Enicurus maculatus			Nepal; Vietnam		KJ456268	KJ455423		KJ454800	KJ455785		
Enicurus schistaceus			Nepal - Ilam		KJ456269	AY878287		GU358727	GU358856	GQ369625	
Enicurus scouleri					KJ456270	KJ455424			HM633716		HM633437
Eumyias [Rhinomyias] additus					HM633371			HM633653	HM633783		HM633512
Eumyias albicaudatus					CU25	CU25		CU26		CU25	CU25
Eumyias indigo					HM633299	FJ173369		HM633581	HM633721		HM633442
Eumyias panayensis					HM633300	FJ173382		HM633582			
Eumyias thalassinus			Nepal - Malde		KJ456274	KJ455430		GU358728	GU358858	GU358993	HM633483
Ficedula [Muscicapella] hodgsoni			India, Arunachal Pradesh		KJ456353	KJ455516		KJ454842	KJ455841	KJ455089	KJ454713
Ficedula hyperythra			India - Arunachal Pradesh		KJ456275	KJ455431		KJ454803	KJ455790	KJ455039	HM633444
Ficedula sapphira			Nepal - Ilam		KJ456276	GU358800		GU358732	GU358862	GU358997	
Ficedula strophiata			India		KJ456277	KJ455432		HM633590	HM633727		HM633450

**Species**	**Sample Number**	**Voucher no**	**Locality**	**Gene**							
				ND3	CYTB	ND2	CO1	MYO	ODC	GAPDH	LDH
Ficedula subrubra			India - Jammu & Kashmir		KJ456278	KJ455433		KJ454804	KJ455791		
Ficedula superciliaris			India - Jammu & Kashmir		KJ456279	KJ455434		KJ454805	KJ455792		HM633449
Ficedula tricolor			India - Arunachal Pradesh		KJ456280	KJ455435		HM633591	KJ455793	KJ455040	HM633451
Ficedula westermanni			Indonesia - Sulawesi		KJ456281	KJ455436		GU358733	GU358863	GU358998	
Ficedula zanthopygia				NC_015802	NC_015802	NC_015802	NC_015802				
Geokichla [Zoothera] citrina			China - Hainan		KJ456515	KJ455701		GU358772	GU358903	GU359038	
Geokichla [Zoothera] wardii			Sri Lanka - Boragasketiya		EU874413	EU874429		KJ454943	KJ455960		
Larvivora [Luscinia] brunnea			India - Jammu & Kashmir		KJ456328	KJ455485		HM633597	HM633734		HM633457
Luscinia [Hodgsonius] phaenicuroides			Nepal - Humla Distr.		KJ456307	KJ455464		GU358736	GU358866	GU359001	HM633455
Luscinia svecica			Russia - Kirovskaya Oblast		HM633323	KJ455487		KJ454828	KJ455826		HM633465
Monticola cinclorhynchus			Nepal – Kipsung		HM633328	KJ455505					HM633470
Monticola rufiventris					GU237082	EF434551		HM633611			HM633471
Monticola solitarius					KJ456346	KJ455506		GU358742	GU358872	GU359007	HM633472
Muscicapa ferruginea			India - Arunachal Pradesh		HM633334	KJ455513		KJ454840	KJ455839	KJ455086	HM633476
Muscicapa latirostris			China – Hebei (nd2)		KJ456350	KJ455512		KJ454839	KJ455838	KJ455085	KJ454712
Muscicapa sibirica			India - Jammu & Kashmir		KJ456352	KJ455515		KJ454841	KJ455840	KJ455088	HM633481
Myadestes obscurus					AF295080	DQ469608					
Myadestes unicolor			Mexico - Veracruz		AF295086	DQ469613		HM633626	HM633757	KJ455091	KJ454715
Myophonus caeruleus			Nepal - Gothichaur valley		KJ456359	KJ455521		GU358750	HM633758	GU359014	
Niltava davidi					HM633352			HM633634	HM633765		HM633493
Niltava grandis			Myanmar - Chin State		KJ456362	KJ455524		KJ454845	KJ455843	KJ455094	HM633494
Niltava macgrigoriae			India - Arunachal Pradesh		KJ456363	KJ455525		KJ454846	KJ455844		HM633495
Niltava sundara			Nepal - Bhojpur Distr.		KJ456364	KJ455526		GU358753	GU358883	GU359018	HM633496
Niltava vivida					HM633356			HM633638	HM633769		HM633497
Oenanthe albonigra					GU055468	GU055420					
Oenanthe deserti					GU237095	GU237121		GU358754	GU358884	GU359019	
Oenanthe picata					GU055487	GU055439					
Oenanthe pleschanka					EU154593	GU055441		EU154705	EU154819		
Phoenicurus [Chaimarrornis] leucocephalus			Nepal - Humla Distr.		KJ456223	KJ455369		GU358714	GU358842	GU358977	HM633416
Phoenicurus [Rhyacornis] fuliginosus			China - Sichuan		HM633372	KJ455627		GU358761	GU358891	GU359026	HM633513
Phoenicurus caeruleocephala			India - Himachal Pradesh		KJ456385	KJ455550					
Phoenicurus erythrogastrus			Captivity		KJ456386	KJ455551		GU358756	GU358886	GU359021	
Phoenicurus frontalis			Nepal - Rasuwa Distr.		KJ456387	KJ455552		HM633645	HM633775		HM633504
Phoenicurus ochruros			China - Qinghai		HM633365	KJ455553		HM633647	HM633777		HM633506
Phoenicurus schisticeps			Captivity		KJ456388	KJ455554			KJ455862		KJ454721
Saxicola caprata			Indonesia - Sulawesi		KJ456454	KJ455631		HM633655	HM633785	KJ455165	HM633514
Saxicola ferreus			India - Uttar Pradesh		KJ456455	KJ455632		GU358762	GU358892	GU359027	HM633515
Saxicola jerdoni			Thailand - Chiang Rai		EU427504	KJ455633					
Saxicola leucurus			India - Punjab		HM633375	KJ455634		HM633657	HM633786		HM633516
Saxicola torquatus			Nepal - Kipsung		KJ456456	KJ455635		KJ454901	KJ455912	KJ455166	HM633517
Tarsiger chrysaeus			India - Himachal Pradesh		HM633387	KJ455663		HM633668	HM633797		HM633528
Tarsiger hyperythrus			Nepal – Rupchet		HM633389	KJ455664		HM633670	HM633799		HM633530
Tarsiger indicus			China - Yunnan		HM633390	KJ455665		HM633671	HM633800		HM633531
Tarsiger rufilatus			India; China		KJ456484	KJ455666		KJ454922	KJ455934	KJ455193	KJ454734
Turdus albocinctus					EU154601	DQ911064		EU154713	EU154828		
Turdus boulboul					EU154606	DQ911068		KJ454939	EU154833		
Turdus maximus					EU154635	KJ455692		EU154746	EU154861		
Turdus rubrocanus					KJ456507	KJ455693		EU154561	EU154896		
Turdus unicolor			Nepal – Kipsung		EU154679	KJ455694		EU154792	EU154907		
Turdus viscivorus					EU154680	DQ911121		EU154793	EU154908	GU359037	
Zoothera dauma			China - Hebei		KJ456516	KJ455702		GU358773	GU358904	GU359039	
Zoothera dixoni					EU154685	AY752320		EU154798	EU154913		
Zoothera marginata					AY752367	AY752325		KF269265	KF269275		
Zoothera mollissima			Nepal - Gonga		KJ456517	KJ455703		KF269266	KF269276	KJ455218	
Zoothera monticola			Nepal - Parbat Distr.		KJ456518	KJ455704		EU154800	EU154915	KJ455219	

Table S3. Morphometric variation in *Montecincla* species

Species & Region	N	Tarsus (Mean, range)	Bill (Mean, range)	Wing (Mean, range)	Tail (Mean, range)	Weight (Mean, range)
M. jerdoni – [A]	21	31.58 (30.27 - 33.29)	21.48 (18.2 - 24.16)	82.87 (69.00– 86.00)	88.22 (74.33 – 93.00)	41.14 (36.00– 48.00)
M. cachinnans – [B]	32	31.87 (27.00 - 36.83)	20.94 (18.59 - 23.67)	85.85 (80.00 - 94.3)	92.75 (70.00 - 100.33)	43.54 (37.00– 58.00)
M. fairbanki – [C]	103	33.31 (29.12 - 36.82)	22.07 (16.57 - 26.27)	86.05 (80.00 - 92.67)	89.34 (59.00 - 98.33)	44.34 (38 – 59.00)
M. meridionalis – [D]	38	34.30 (31.04 - 36.71)	23.34 (19.71 - 26.62)	87.82 (82.00– 94.00)	91.18 (84 - 110)	51.42 (44.00– 59.00)

Table S4. Morphometric variation in *Sholicola* species

Species & Region	N	Tarsus (mean, range)	Wing (mean, range)	Tail (mean, range)	Bill (mean, range)	Weight (mean, range)
S. major – [A]	45	27.23 (19.47-31.10)	79.86 (70.33 – 86.00)	64.39 (59.00– 79.00)	17.85 (14.58 - 19.74)	24.97 (17.75 – 29.00)
S. major – [B]	42	27.86 (21.67-31.67)	81.12 (74.67 - 89.33)	64.63 (58.00– 81.00)	17.43 (13.15 - 21.33)	24.84 (20.00– 29.00)
S. albiventris – [C]	76	28.22 (25.66 - 31.36)	76.95 (69.67 – 84.00)	63.35 (56.00– 79.00)	17.40 (13.32 - 20.08)	22.87 (17.00– 32.00)
S. ashambuensis – [D]	21	26.95 (24.7 - 28.5)	76.16 (71.00– 83.00)	60.73 (52.00– 68.00)	18.12 (16.5 - 19.64)	24.07 (18.19 - 27.50)

Table S5. ***Montecincla*** **song variation in the Western Ghat Sky Islands (mean, SE, range)**

	M. jerdoni **N=7**	***M. cachinnans*** **N=3**	***M. fairbanki*** **N=6**	***M. meridionale*** **N=7**
**No. notes per song**	9.42, 2.78, 3-25	11, 2.5, 8-16	6.34, 1.45, 3-13	11.42, 3.27, 3-27
**Note length (seconds)**	0.1609, 0.008, 0.01-0.39	0.19, 0.009, 0.06-0.35	0.27, 0.01, 0.08-0.52	0.23, 0.006, 0.12-0.49
**Song bandwidth (kHz)**	0.80, 0.02, 0.56-1.46	0.91, 0.19, 0.59-1.48	0.95, 0.03, 0.61-1.46	0.61, 0.008, 0.39-0.97
**Song delivery rate (notes/second)**	2, 0.86, 1-4	2.34, 0.6, 1-4	1.5, 0.32, 1-2	2.57, 0.2, 1-5
**Phrase length seconds**	5.97, 1.11, 0.1-34.93	1.84, 0.32, 0.2-8.513	0.785, 0.1, 0.72-2.79	2.14, 0.27, 0.3-13.53

Table S6. ***Sholicola*** **song variation in the Western Ghat Sky Islands (mean, SE, range)**

	S. major	***S. albiventris***	***S. ashambuensis***
**No. notes per song**	6.7, 0.25, 3-17	13.3, 0.57, 3-34	9.3, 0.36, 2-32
**Note length (seconds)**	0.36, 0.01, 0.19-0.62	0.21, 0.01, 0.09-0.4	0.25, 0.01, 0.08-0.35
**Song bandwidth (kHz)**	3.6, 0.12, 0.81-6.7	5.5, 0.01, 2.3-7.9	4.9, 0.01, 2.6-6.9
**Song delivery rate (notes/second)**	2.8, 0.08, 0.04-5.0	4.7, 0.42, 2.6-7.8	4.2, 0.12, 0.94-7.6
**Phrase length (seconds)**	2.5, 0.10, 1.2-5.8	2.9, 0.10, 0.8-6.3	2.4, 0.13, 0.8-6.7

Table S7. Comparison of morphometric measurements of *Sholicola ashambuensis* and *S. albiventris*

	Sholicola albiventris			*Sholicola ashambuensis*		
	N	Mean	**Std Dev**	**N**	**Mean**	**Std Dev**
**Tarsus**	76	28.22	1.04	21	26.95	0.93
**Wing**	76	76.95	3.21	21	76.16	3.45
**Bill**	76	17.40	1.49	21	18.12	0.74
**Tail**	75	63.35	4.17	21	60.73	4.23

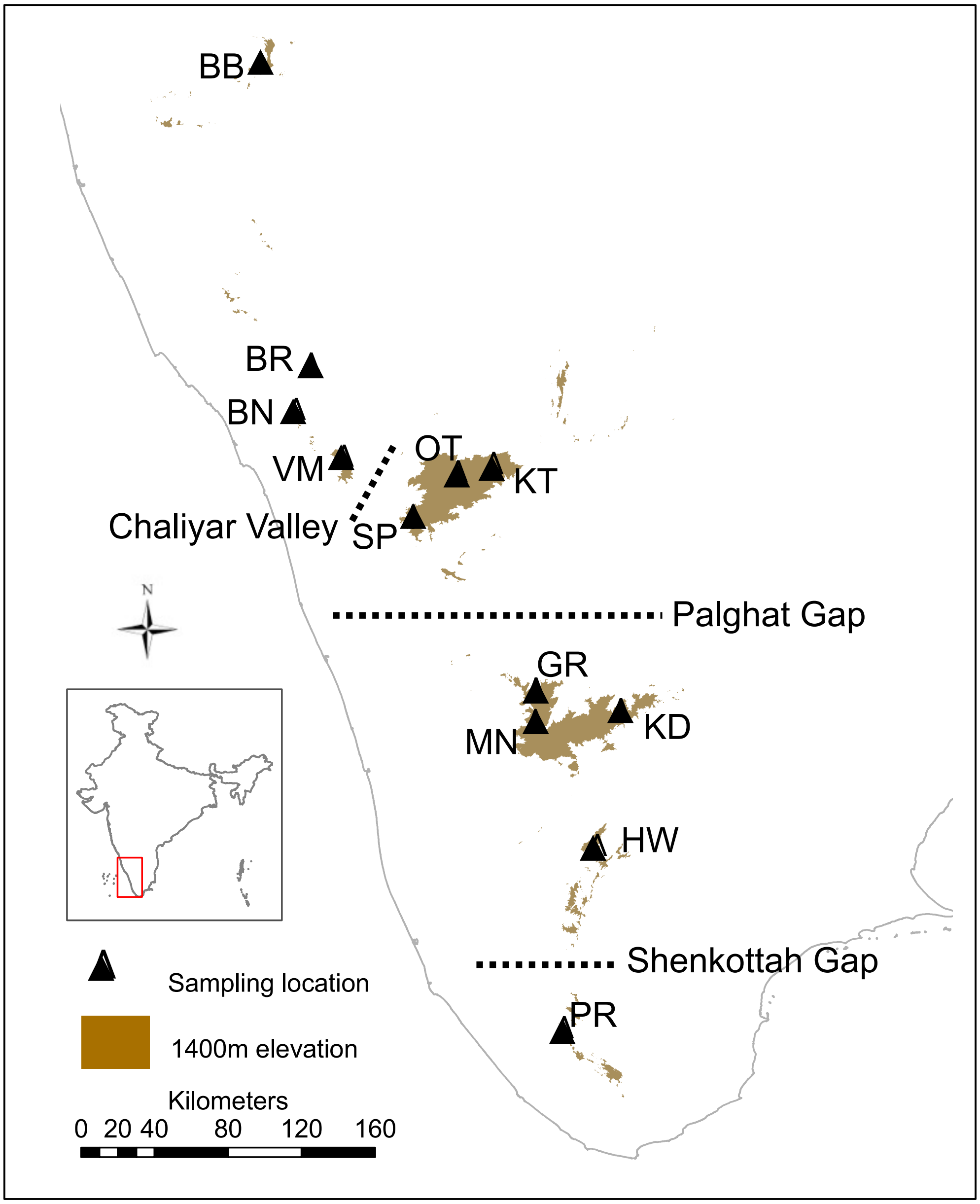

Figure S1. Sample collection sites across the sky islands of the Western Ghats. Two letter codes refer to: BB- Bababudan Hills; BR – Brahmagiris; BN – Banasura; VM – Vellarimala; SP – Sispara; OT – Ooty; KT – Kotagiri; GR – Grasshills; MN – Munnar; KD – Kodaikanal; HW - Highwavies (Meghamalai); PR - Peppara (Ashambu hills).

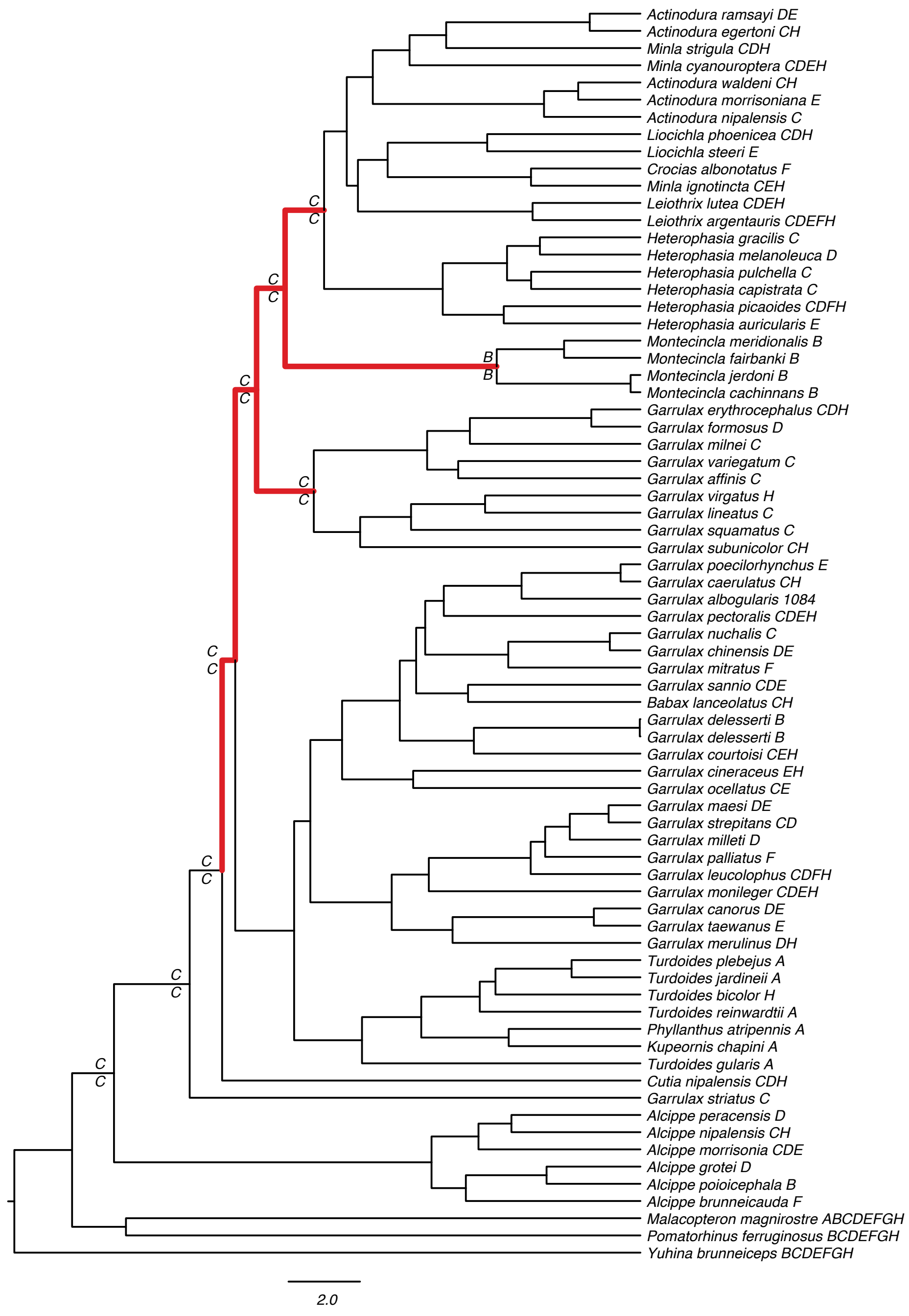

Figure S2. Ancestral area analysis using Lagrange for *Montecincla.* Area codes for terminal taxa are A-Africa, B-peninsular India, C-Himalayas, D-Southeast Asia, E-China, F-Sundaland, G-Philippines, and H-Assam. The most likely ancestral range reconstruction for relevant nodes (in red) are shown.

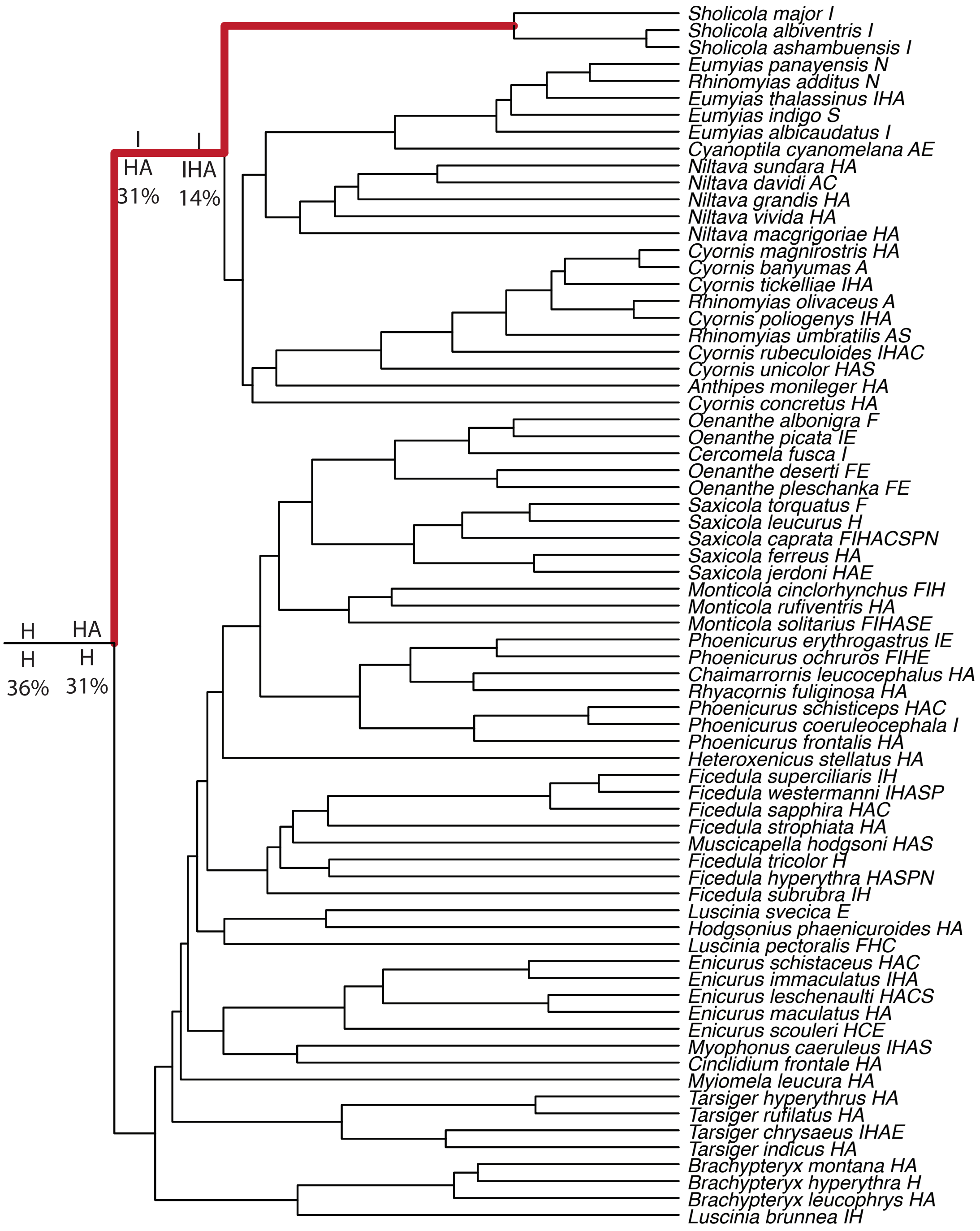

Figure S3. Ancestral area analysis using Lagrange for *Sholicola*. Area codes for terminal taxa are I-Peninsular India, H-Himalayas, A-Southeast Asia, D-Eurasia, E-Australasia F-Africa + Southwest Asia, C-China, S-Sundaland, P-Philippines, E-Eurasia, and N-Australasia/New World. The most likely ancestral range reconstruction for relevant nodes (in red) are shown with their relative probabilities.

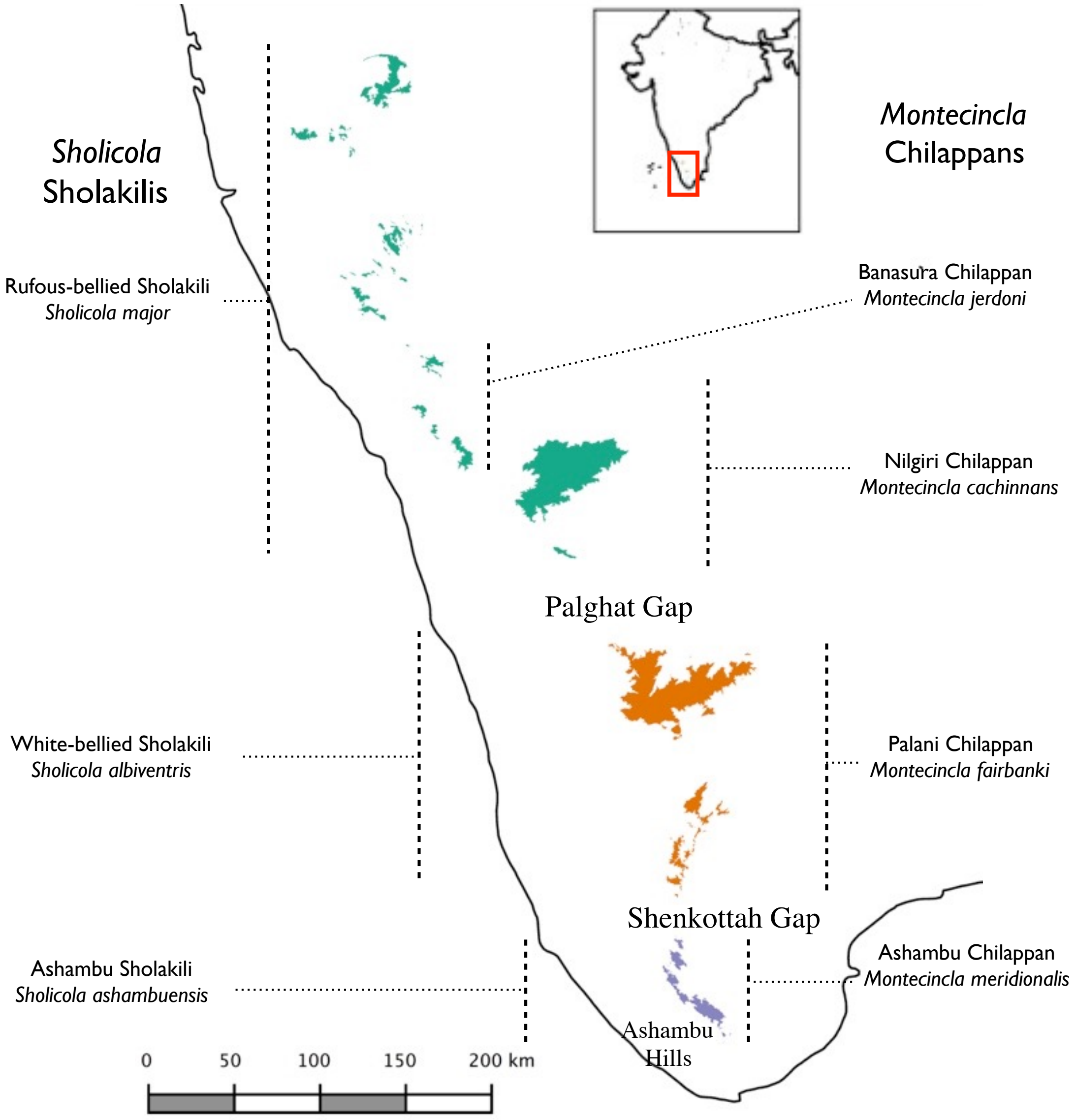

Figure S4. Map showing the allopatric distribution of *Montecincla* chilappans and *Sholicola* sholakilis.

a)

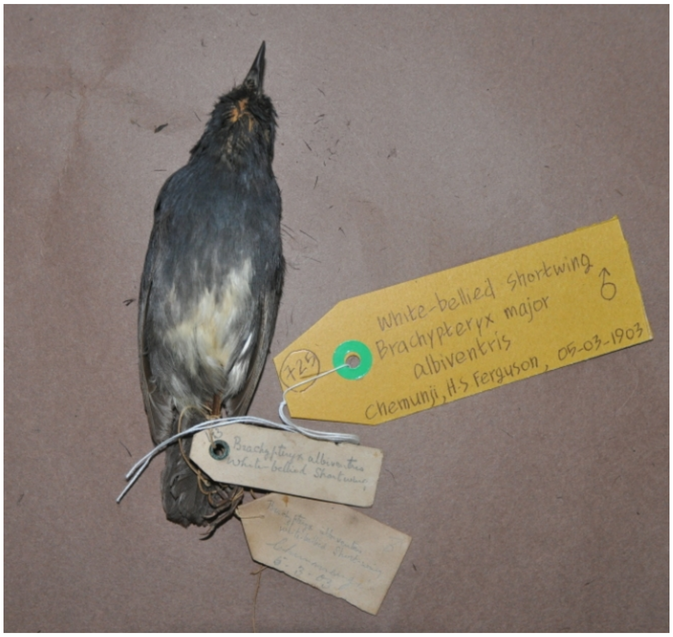

b)

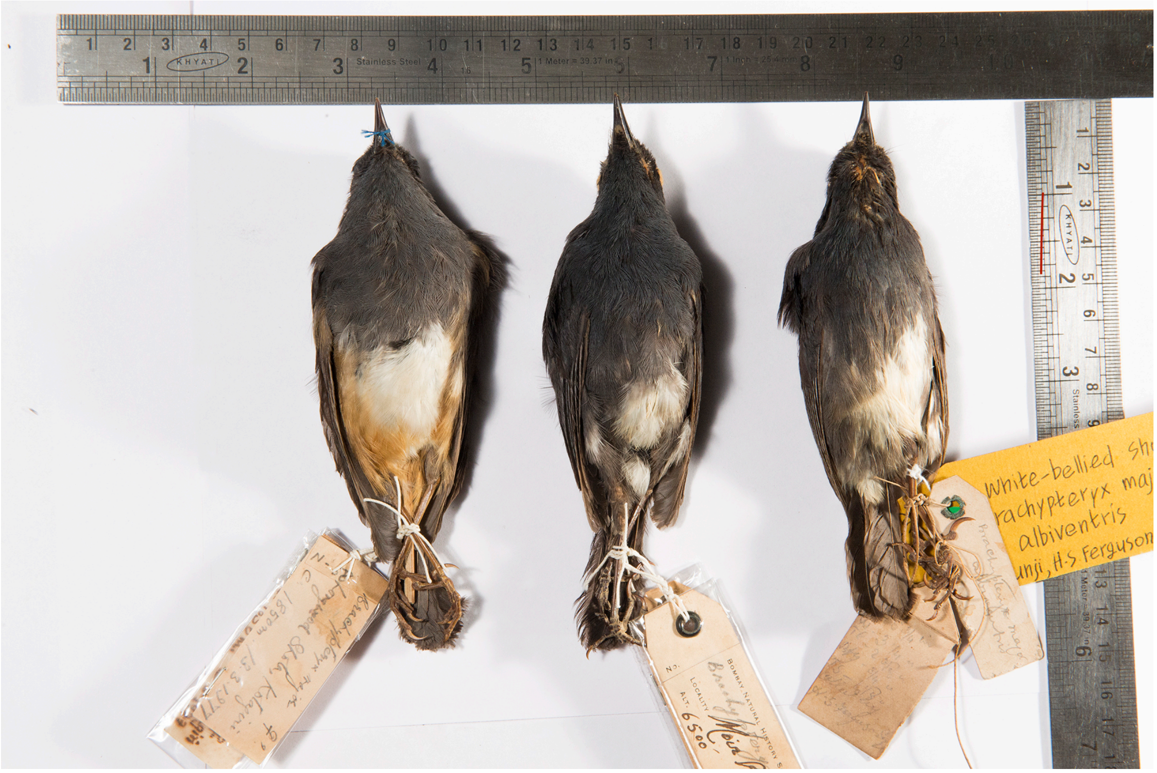

Figure S5. Photographs of *Sholicola ashambuensis sp. nov.*; a) holotype in the Trivandrum Museum of Natural History; b) in comparison with other members of *Sholicola*: *S. ashambuensis* (right), with larger white belly contrasted with *S. albiventris* (middle), *S. major* (left).

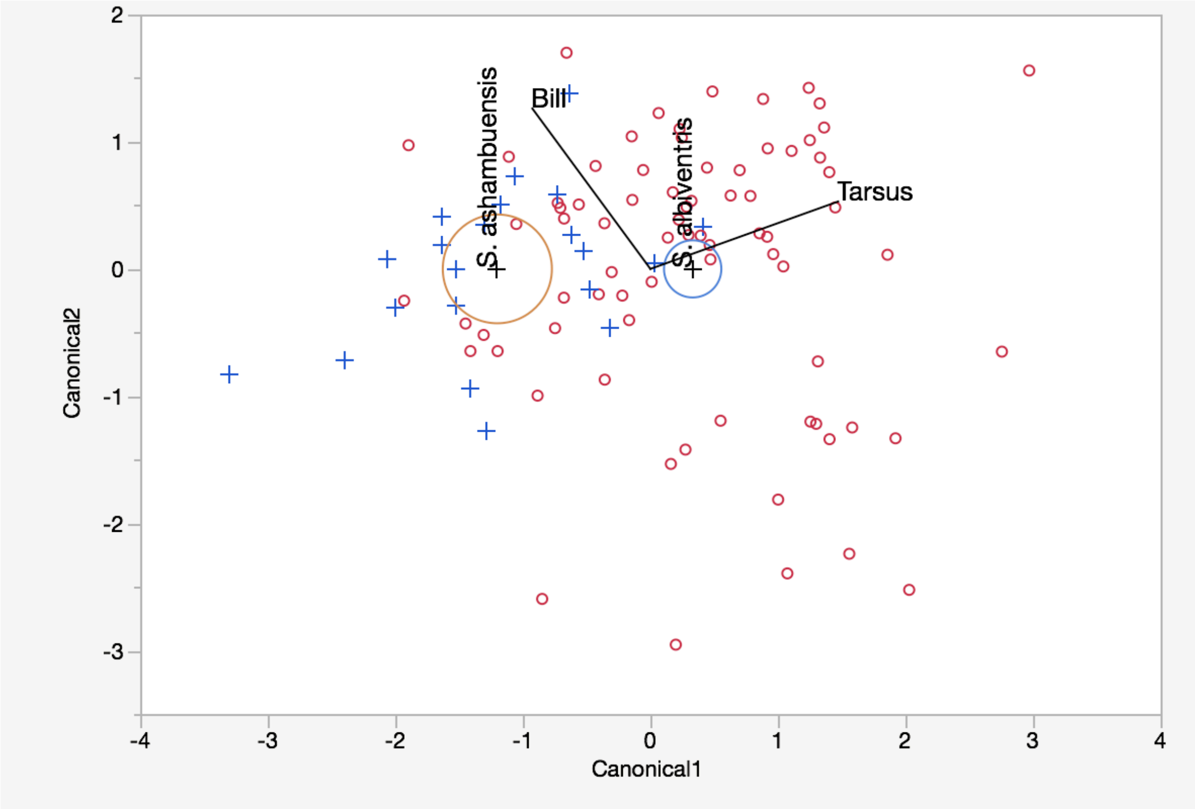

Figure S6. a) Discriminant Function Analysis of morphometric differences between *Sholicola ashambuensis* and *S. albiventris* based on 97 individuals shows 79.4% accurate classification and significant differences in the means (non overlapping 95% confidence interval circles).

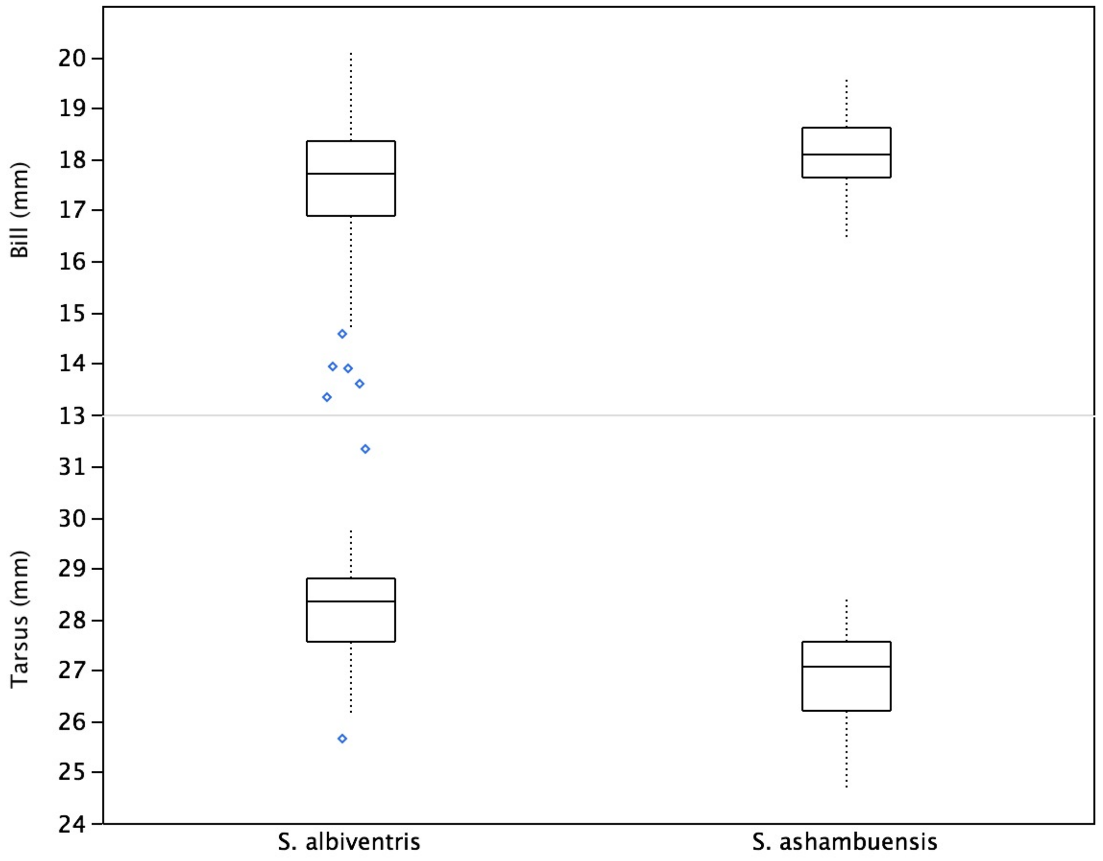

Figure S6. **b**) Box plots showing morphometric differences in bill and tarsus lengths in *Sholicola ashambuensis* and *S. albiventris*.

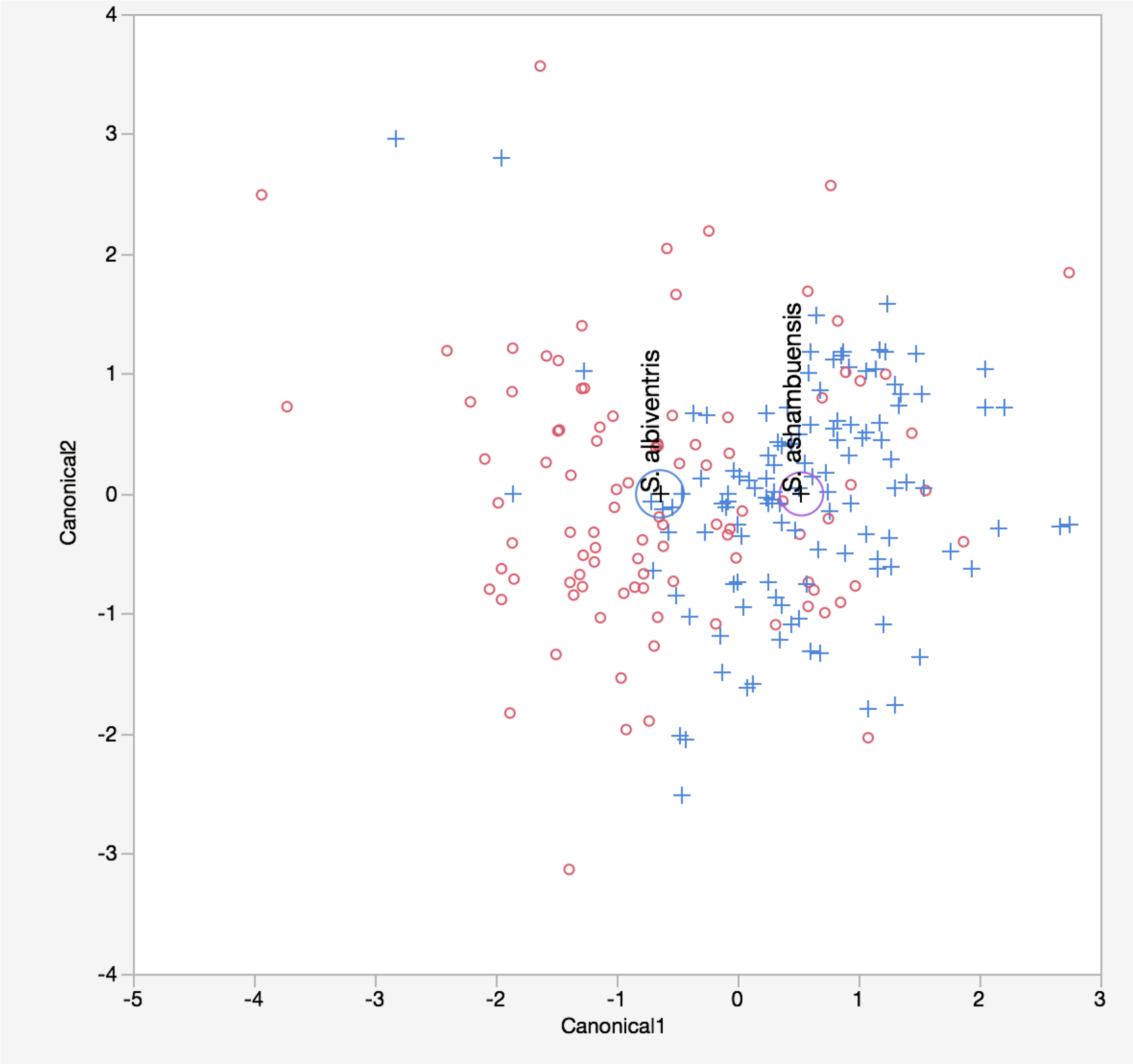

Figure S7. Discriminant Function Analysis of *Sholicola ashambuensis* and *S. albiventris* songs based on 217 song recordings shows 80% accurate classification and significant differences in the means (non-overlapping 95% confidence interval circles).
